# BDNF–TrkB signaling in striatopallidal neurons controls inhibition of locomotor behavior

**DOI:** 10.1038/ncomms3031

**Published:** 2013-06-18

**Authors:** Dario Besusso, Mirjam Geibel, Dana Kramer, Tomasz Schneider, Valentina Pendolino, Barbara Picconi, Paolo Calabresi, David M. Bannerman, Liliana Minichiello

**Affiliations:** 1 Centre for Neuroregeneration, University of Edinburgh, EH16 4SB Edinburgh, UK; 2 Mouse Biology Unit, European Molecular Biology Laboratory, Via Ramarini 32, 00015 Monterotondo, Italy; 3 Department of Pharmacology, University of Oxford, Oxford OX1 3QT, UK; 4 Department of Experimental Psychology, University of Oxford, Oxford OX1 3UD, UK; 5 Fondazione Santa Lucia, Istituto di Ricovero e Cura a Carattere Scientifico, 00179 Rome, Italy; 6 Clinica Neurologica, University of Perugia, Ospedale S. Maria della Misericordia, 06156 Perugia, Italy

## Abstract

The physiology of brain-derived neurotrophic factor signaling in enkephalinergic striatopallidal neurons is poorly understood. Changes in cortical *Bdnf* expression levels, and/or impairment in brain-derived neurotrophic factor anterograde transport induced by mutant huntingtin (m*Hdh*) are believed to cause striatopallidal neuron vulnerability in early-stage Huntington’s disease. Although several studies have confirmed a link between altered cortical brain-derived neurotrophic factor signaling and striatal vulnerability, it is not known whether the effects are mediated via the brain-derived neurotrophic factor receptor TrkB, and whether they are direct or indirect. Using a novel genetic mouse model, here, we show that selective removal of brain-derived neurotrophic factor–TrkB signaling from enkephalinergic striatal targets unexpectedly leads to spontaneous and drug-induced hyperlocomotion. This is associated with dopamine D2 receptor-dependent increased striatal protein kinase C and MAP kinase activation, resulting in altered intrinsic activation of striatal enkephalinergic neurons. Therefore, brain-derived neurotrophic factor/TrkB signaling in striatopallidal neurons controls inhibition of locomotor behavior by modulating neuronal activity in response to excitatory input through the protein kinase C/MAP kinase pathway.

## Abstract (web summary)

The neurotrophic factor BDNF is implicated in striatal cell long-term survival. Besusso *et al*. selectively delete BDNF receptors in the striatal circuitry of mice and find that this leads to hyperlocomotion, which is associated with dopamine receptor-dependent increases in specific kinases.

## Graphical abstract

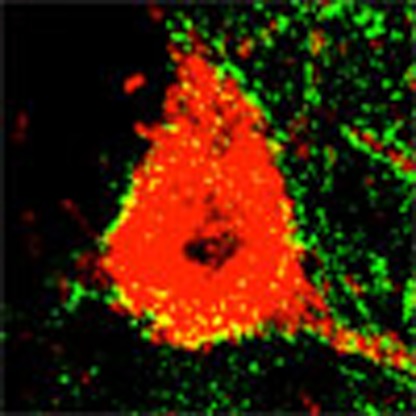

Distinct basal ganglia (BG) circuits involved in motor control (direct and indirect pathways), originate in the dorsal striatum. These receive convergent excitatory afferents from the cortex and thalamus. The direct striatonigral pathway activity induces disinhibition of excitatory thalamocortical projections and facilitation of movement. The indirect striatopallidal pathway induces inhibition of thalamocortical projection neurons, which decrease cortical premotor drive and inhibit movement^1^,^2^. Preferential loss of the indirect pathway medium-sized spiny neurons (MSNs) is thought to decrease the amount of inhibitory control over unwanted movements, leading to chorea and hyperkinesia typically associated with define HD: Huntington's disease (HD) 3(ref 3). HD is an autosomal dominant and progressive neurodegenerative disorder characterized by severe deficits in motor function, cognitive and psychiatric changes. This disorder is correlated with an expansion of a CAG repeat at the N-terminal region of the huntingtin gene^4^.

Although the exact mechanism by which m*Hdh* causes neuronal dysfunction is not yet established, it is clear that regions of the brain such as the striatum are the first to degenerate.

Previous studies at early stages of the disease demonstrate that striatal enkephalinergic (ENK)-positive GABAergic medium spiny neurons projecting to the external part of the globus pallidus are more vulnerable than the striatal MSN subset expressing substance P, an effect also found in HD mouse models^5^,^6^,^7^,^8^. These neurons, also known as striatopallidal neurons, coexpress the dopamine receptor 2 (D_2_R)^9^. Their degeneration in HD ultimately results in the loss of inhibitory input to the thalamus, enhancing involuntary movements (chorea)^10^. The mechanisms that drive selective striatal cell loss are currently unclear and still extensively investigated. A recent hypothesis suggests that m*Hdh* drives a progressive decline in striatal trophic support that ultimately leads to MSNs degeneration, initially of ENK–MSNs^11^. The neurotrophic brain-derived neurotrophic factor (BDNF) appears to be indispensable for striatal cell long-term survival and maintenance. Cortical neurons are the main source of striatal BDNF, which is delivered by anterograde transport through the corticostriatal afferents^12^. Mutant *Hdh* impairs BDNF promoter II transcriptional activity through the cytoplasmic retention of the neuron-specific transcriptional repressor REST^11^, and can also interfere with BDNF anterograde transport from cortical neurons to striatal targets^13^. Therefore, reduction in BDNF supply to the striatum jeopardizes the long-term survival and morphology of striatal MSNs^14^,^15^, contributing ultimately to striatal degeneration in HD. This is consistent with the reduced cortical and striatal BDNF levels observed in both HD patients and mouse models^16^. Forebrain-specific *Bdnf* mutants recapitulate many behavioral and anatomical abnormalities seen in mouse models of HD^14^. Similarly, mice carrying m*Hdh* transgene, with genetic reduction of BDNF levels, exhibit advanced onset and increased severity of motor dysfunction, resulting from the degeneration of striatal ENK–MSNs^5^.

Despite extensive evidence associating cortical *Bdnf* levels with striatal vulnerability, there is no demonstration that these effects are mediated *via* TrkB. This is mainly due to any ablation or alteration of *Bdnf* levels, either globally or in a region-specific manner, affecting cortical physiology^17^, and therefore confounding the action of *Bdnf* anterogradely transported from the cortex to other regions of the brain, such as its striatal targets.

To address the physiological role of BDNF/TrkB signaling in striatopallidal neurons, we have selectively deleted the high-affinity BDNF receptor, TrkB, from ENK^+^MSNs. Surprisingly, this manipulation led to spontaneous and drug-induced hyperlocomotion associated with increased D2R-dependent MAPK/PKC phosphorylation and reduced striatopallidal activation, without obvious effects on motor coordination and gait parameters, or long-term survival and morphological defects of ENK^+^MSNs. We therefore demonstrate that BDNF–TrkB signaling in striatal ENK^+^MSNs contributes to the inhibitory control of locomotor behavior exerted by the indirect pathway.

## Results

### Generation of Enkephalin-specific *Trkb* knockout mice

**Figure 1:**
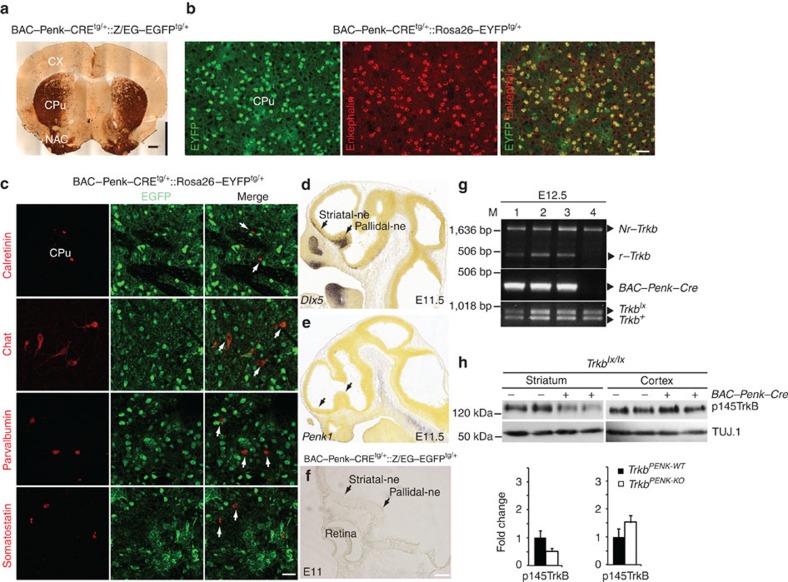
Generation of *Trkb-enkephalin* knockout mice. (**a**) Adult brain image of *BAC–Penk–Cre*^*tg/+*^*::Z/EG–egfp*^*tg/+*^ mouse stained with G(Y)FP Abs revealing *Cre*-mediated GFP expression in specific brain areas, the caudate-putamen (CPu), the nucleus accumbens (NAc), scattered cells in cortical layer II and V–VI (CX). (**b**) Confocal images showing *Cre*-mediated EYFP expression in ENK striatal cells, (**c**) and absence of EYFP expression in striatal interneurons (arrows) identified by specific markers. (**d,e**) *Dlx5 in situ* expression at E11.5 in the striato-pallidal neural ephitelium (stiatal-ne, pallidal-ne), and absence of Penk1 expression in the same regions at this stage (images are from the Allen Mouse Brain Atlas^20^). (**f**) E11 *BAC–Penk–Cre*^*tg/+*^*::Z/EG–egfp*^*tg/+*^ transversal brain section with no GFP staining in the striato-pallidal neural ephitelium. (**g**) PCR analysis of *Trkb* recombination in brains carrying the *BAC–Penk–Cre* transgene at E12.5. Primers were specific for un-recombined (Nr) and recombined (r) *Trkb*^*lx*^ allele; each embryo carried a *Trkb*^*lx*^ allele and *Trkb*^*WT*^ allele. Recombination occurred only in presence of the *Cre* transgene (embryos 1–3). (**h**) Protein lysates from different brain regions of adult *Trkb*^*lx/lx*^ mice either in presence (+) or in absence (−) of the *Cre* transgene were analyzed for TrkB protein levels. Blots were probed with Abs to p145-TrkB. Striatal TrkB levels were 50% reduced, but unchanged in the cortex. Blots were reprobed with Abs to TUJ.1 to control for loading and quantification of fold changes (values are mean±s.d.). ChAT, choline acetyltransferase. Scale bars: **a**, 500 μm; **b,c**, 50 μm; **f**, 100 μm. See also Supplementary Fig. S1.

To address the physiological relevance of BDNF–TrkB signaling in the ENK^+^MSNs, we conditionally deleted *Trkb* from these neurons by generating a BAC transgenic mouse line carrying *Cre*-recombinase under the control of the pre-proenkephalin promoter (BAC–*Penk–Cre*^*tg/+*^) (Supplementary Fig. S1a). We studied the recombination pattern of the BAC–*Penk–Cre* line using two reporter lines^18^,^19^ (Fig. 1a–c), which recapitulated mostly the expression of the pre-proenkephalin gene reported in literature^20^. Namely, recombination was detected in particular in the caudate-putamen, the nucleus accumbens, scattered cells in cortical layer II and V–VI (Fig. 1a), and the granular layer of the olfactory bulb and cerebellum. The cellular specificity of *Cre*-recombination was examined by double immunofluorescence using antibodies (Abs) to YFP and to markers either specific for ENK^+^MSNs, showing *Cre*-mediated EYFP expression occurring in 98% of the ENK^+^MSNs (Fig. 1b), or specific for striatal interneurons, showing no colocalisation (Fig. 1c). The relative abundance of EYFP^+^MSNs within the striatum was estimated using COUPTF1-interacting protein 2 (CTIP2) (ref. 21). We found 40–50% EYFP^+^MSNs (Supplementary Fig. S1b), consistent with the literature^22^. However, 4% of the total EYFP^+^ cells do not express enkephalin. Possibly the enkephalin-promoter was active at some point during cell differentiation or expression of enkephalin is below the minimum detectable level. This novel *Cre* line was crossed to a *Trkb* floxed line^23^ to generate *Trkb*^*PENK-KO*^ mice. E(Y)GFP immunoreactivity was not detected in the developing mouse brain before E12.5, consistent with enkephalin expression in differentiating multipotent progenitors but not in earlier progenitors, unlike *Dlx5* (refs. 20, 24) (Fig. 1d–g). Western blot analysis of adult mouse brain lysate further confirmed specific striatal TrkB reduction in *Trkb*^*PENK-KO*^ mice compared with *Cre*-negative *Trkb*^*PENK-WT*^ littermates (*Trkb*^*lx/lx*^;*PENK*^*+/+*^) (Fig. 1h). *Trkb* deletion was also validated by *in situ* hybridization combined with immunofluorescence double staining (*Trkb*/EYFP), (Supplementary Fig. S1c). These data confirmed the specificity and successful inactivation of *Trkb* in ENK^+^ neurons.

### *Trkb* deletion in ENK MSNs results in hyperlocomotion

**Figure 2:**
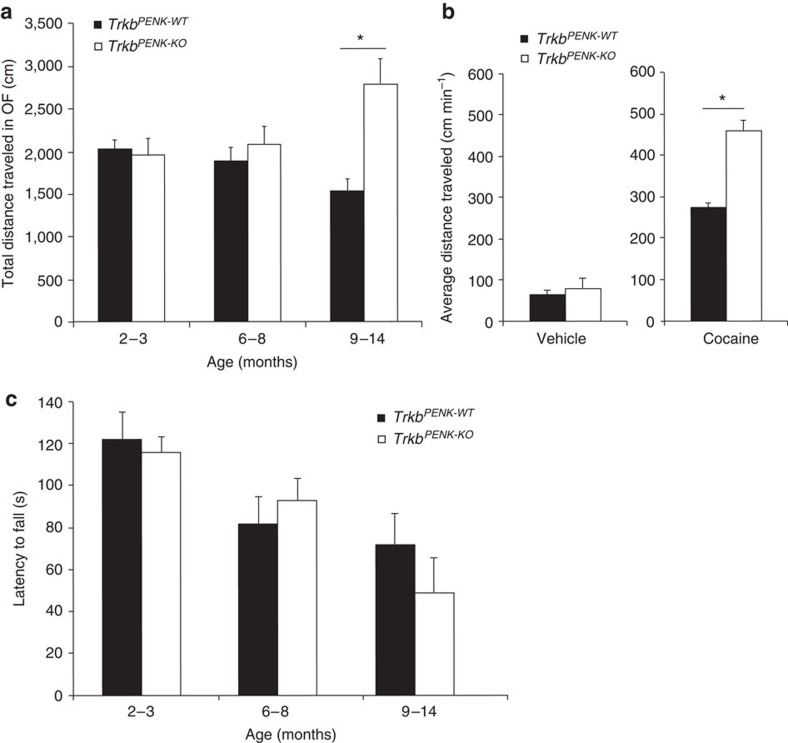
Enhanced spontaneous and cocaine-induced locomotion in *Trkb*^*PENK-KO*^ mice. (**a**) Spontaneous locomotor measures obtained for control and mutant mice tested as independent groups at different stages (*Trkb*^*PENK-WT*^, *n*=9, 9, 7; *Trkb*^*PENK-KO*^, *n*=7, 9, 7, for the three groups, respectively). Mice were individually placed into an OF arena for 30 min and the distance traveled was recorded. While no difference was observed up to 8 M of age, a sharp significant increase in total distance traveled was found in *Trkb*^*PENK-KO*^ mice compared with that in *Trkb*^*PENK-WT*^ littermates by 9 M of age until later in life (two-way ANOVA, *F*_(1,42)_=8.964; *P*=0.0046, main effect of genotype; *F*_(2,42)_=6.406; *P*=0.0037, interaction between genotype and age, *Trkb*^*PENK-KO*^ versus *Trkb*^*PENK-WT*^, *P=0.0001); values are means±s.e.m. (**b**) Cocaine-induced hyperlocomotion. After 2 days of habituation to the apparatus, mice (*Trkb*^*PENK-WT*^, *n*=11; *Trkb*^*PENK-KO*^, *n*=10) were i.p.-injected with either vehicle on day 0, showing no difference between mutants and controls (one-way ANOVA, *F*_(1,19)_=0.386; *P*=0.5417), or with 10 mg kg^−1^ cocaine for 3 consecutive days, immediately before being placed into the arena where locomotor activity was recorded for 30 min (one-way ANOVA, *F*_(1,19)_=4.797; *P=0.0412). Cocaine values are mean across the 3 days±s.e.m. (**c**) *Trkb*^*PENK-KO*^ and *Trkb*^*PENK-WT*^ mice were tested for motor coordination as independent groups at different stages (*Trkb*^*PENK-WT*^, *n*=9, 8, 8, and *Trkb*^*PENK-KO*^ mice, *n*=7, 11, 7, for the three groups, respectively). On day 1, they were habituated to the rotarod apparatus and subjected to three sessions of 3 min at 4 r.p.m. constant speed, separated by 15-min rest intervals. The next day they were placed on the rod accelerating from 4 to 40 rpm for three consecutive sessions (separated by 5-min intervals). Latency of each animal to fall from the rod was scored. The performance was not significantly different between mutants and controls (two-way ANOVA, *F*_(1,44)_=0.298; *P*=0.5878 main effect of genotype; *F*_(2,44)_=0.874; *P*=0.4244, interaction between genotype and age; *F*_(2,44)_=9.702; *P*=0.0003 effect of age). Values are means±s.e.m.

Recent work, by toxin ablation or functional disruption of the striatopallidal D_2_R neurons, has provided direct evidence that these neurons regulate inhibitory functions on locomotor activity^25^,^26^. To understand if BDNF–TrkB signaling would be relevant to this function, we tested locomotor activity of *Trkb*^*PENK-KO*^ mice and age-matched *Trkb*^*PENK-WT*^ in the open-field (OF) apparatus (Fig. 2a). We found a sharp significant increase in total distance traveled in *Trkb*^*PENK-KO*^ mice compared with that in *Trkb*^*PENK-WT*^ littermates by 9 M of age until later in life (Fig. 2a). The *Trkb*^*PENK-KO*^ spontaneous hyperlocomotion observed in the OF cannot be due to increased anxiety or general activity of the mice, as analysis of time spent in the border compared with that in the center of the OF apparatus, and analysis of home-cage activity over 3 days did not reveal significant differences between genotypes (Supplementary Fig. S2a,b). Mice carrying only the *Cre* transgene tested at 9 M of age in an OF did not show significant changes compared with wild-type mice (*P*=0.897, unpaired Student’s *t*-test), confirming that the spontaneous increased locomotion observed in the *Trkb*^*PENK-KO*^ mice was due to the specific deletion of *Trkb* in ENK^+^MSNs.

Drugs of abuse, like cocaine, are able to induce hyperlocomotion, an action that is largely mediated through the striatum^27^. As the hyperlocomotion phenotype observed in the OF is age-dependent, it is likely that young mice already have a mild defect in motor control that is not detectable in a paradigm of spontaneous locomotion. Therefore, we tested the response of young adult (3 M old) *Trkb*^*PENK-KO*^ and *Trkb*^*PENK-WT*^ mice in the OF when acutely challenged with cocaine. *Trkb*^*PENK-KO*^ mice showed significantly increased distance traveled compared with *Trkb*^*PENK-WT*^ littermates upon cocaine treatment (Fig. 2b), demonstrating that lack of BDNF–TrkB signaling in ENK^+^MSNs enhances cocaine-induced hyperlocomotion. This confirms that striatopallidal neurons lacking BDNF–TrkB signaling lose inhibitory control over locomotor activity, and that young mutants show a phenotype similar to the aged one if challenged.

To assess if lack of TrkB signaling in ENK^+^MSNs would affect motor coordination and overall motor function, we first examined *Trkb*^*PENK-KO*^ mice and *Trkb*^*PENK-WT*^ littermates for clasping, and found normal hind-paw clasping in both genotypes at all stages analyzed. We then assessed motor coordination performance of *Trkb*^*PENK-KO*^ using the rotarod test. Twenty-five *Trkb*^*PENK-WT*^ and 25 *Trkb*^*PENK-KO*^ mice from 2 to 14 M of age were tested. Mutants showed similar performance compared with control littermates (Fig. 2c), indicating that lack of BDNF–TrkB signaling in striatopallidal ENK^+^MSNs does not affect general performance in motor coordination.

### Subtle gait differences in *Trkb*^*PENK-KO*^ mice

As neither the OF nor the rotarod test would allow for objective assessment and quantitative measures of fine motor movements, we then used the CatWalk test; a new method of quantitative gait analysis^28^,^29^. This method allows easy quantification of a large number of locomotion parameters during walkway crossing (Supplementary Methods). This test has also been validated to study motor impairment associated with different neurological motor disorders, including an HD model^30^. Analysis of mice at 5 M of age, before mutants develop spontaneous hyperlocomotion, revealed no significant differences between genotypes in activity-related parameters (Supplementary Table S1). All parameters related to single paws, previously reported to be compromised in a transgenic rat model of HD^30^, were normal in *Trkb*^*PENK-KO*^ mice compared with that in controls (Supplementary Fig. S3a–d, Supplementary Table S2). However, a smaller print width for the left hind paw (LH) was found in mutant mice compared with that in controls (Supplementary Fig. S3e, Supplementary Table S2). There was no difference between genotypes in front-paw parameters (Supplementary Table S2).

As for the coordination-related gait parameters, both mutant and control mice showed normal interlimb coordination based on the analysis of the step sequence patterns, and normal phase dispersion (Supplementary Table S3, Supplementary Fig. S4). However, some differences between genotypes were found in the time relationship between footfalls of two different paws (couplings), with slightly decreased timely coordination between ipsilateral (RH→RF) and girdle paws (RH→LH), but slightly increased coordination between diagonal paws (RF→LH) in *Trkb*^*PENK-KO*^ mice compared with that in controls (Supplementary Fig. S3f-g).

Mice examined in the CatWalk test were additionally analyzed in the inverted screen test, which requires motor coordination and muscle strength, and in the weight-lifting test to further ascertain their strength/forepaw grasping. No significant differences were scored between the two genotypes for either test (Supplementary Fig. S5). Overall, these data reveal subtle changes in gait parameters in *Trkb*^*PENK-KO*^ mice, which are evident before the appearance of spontaneous altered locomotion.

### Reduced ENK expression in striatal MSNs upon *Trkb* deletion

**Figure 3:**
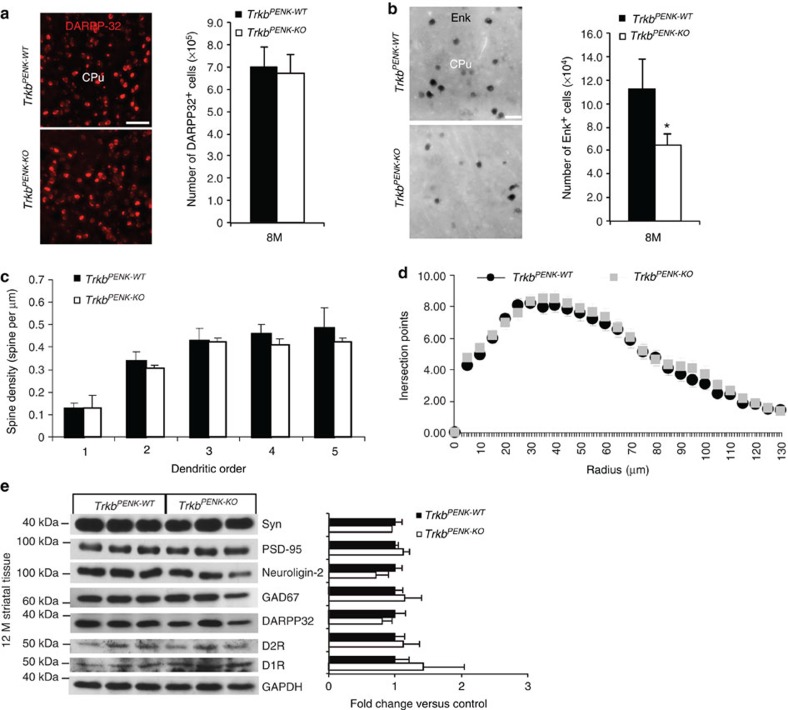
Reduced striatal enkephalin expression in *Trkb*^*PENK-KO*^ mice in the absence of striatal loss. (**a**) Striatal images from 8-M-old mice immunostained for DARPP-32. DARPP-32 cell counts at 8 M (*Trkb*^*PENK-KO*^ *n*=3, versus *Trkb*^*PENK-WT*^ *n*=3, *P*=0.741, unpaired Student’s *t*-test). (**b**) Striatal images of 8-M-old *Trkb*^*PENK-WT*^ and *Trkb*^*PENK-KO*^ mice showing enkephalin *in situ* hybridization. Counts of ENK^+^MSNs at 8 M (*Trkb*^*PENK-KO*^ *n*=3, versus *Trkb*^*PENK-WT*^ *n*=3, *P=0.036, unpaired Student’s *t*-test). (**c**) Confocal stacks of Golgi-impregnated MSNs from 12-M-old mice analyzed for spine density. (**d**) Sholl analysis of striatal MSNs dendritic complexity. For both panels **c** and **d** (*n*=30, 10 randomly selected neurons per mouse/over three mice). One-way ANOVA, *F*_*(1,58)*_=0.341; *P*=0.5615. See also Supplementary Figs S6 and S7. (**e**) Representative western blot of 12-M-old striatal tissue lysates of *Trkb*^*PENK-WT*^ (*n*=3) and *Trkb*^*PENK-KO*^ (*n*=3) mice. Membranes were blotted with the indicated Abs and densitometry analysis was used for quantification of the proteins relative abundance (*Trkb*^*PENK-KO*^ versus *Trkb*^*PENK-WT*^ for Syn, *P*=0.46; PSD-95, *P*=0.15; Neuroligin-2, *P*=0.09; GAD67, *P*=0.40; DARPP-32, *P*=0.21; D2R, *P*=0.52; D1R, *P*=0.33. *P*-values were generated by unpaired Student’s *t*-test). GAPDH was used to control for protein loading. Values are means±s.e.m. See also Supplementary Fig. S8a. CPu, caudate-putamen. Scale bars: **a**–**b**, 50 μm. Syn, synaptophysin; D1R and D2R, dopamine receptor type 1 and 2.

To rule out that the phenotype observed in locomotor activity was not the result of loss or morphological alteration of the ENK^+^MSNs but rather a primary effect of the lack of TrkB signaling in ENK^+^MSNs, we first examined the relative abundance of striatal MSNs in aged *Trkb*^*PENK-KO*^ mice and *Trkb*^*PENK-WT*^ littermates. Counts of neurons labeled with Abs against an MSN-specific marker, like dopamine- and cAMP-regulated phosphoprotein of 32 kDa (DARPP-32), did not reveal loss of striatal MSNs in *Trkb*^*PENK-KO*^ mice compared with *Trkb*^*PENK-WT*^ littermates (Fig. 3a). Stereological analysis of histological sections revealed normal striatal volume in *Trkb*^*PENK-KO*^ compared with that in *Trkb*^*PENK-WT*^ mice at 12 M of age (Supplementary Fig. S6a,b). Computer-based morphometric analysis of Golgi-impregnated striatal MSNs showed no difference in dendritic spine density (Fig. 3c, and Supplementary Fig. S6c) and thickness (Supplementary Fig. S6d) between 12-M old *Trkb*^*PENK-KO*^ mice and *Trkb*^*PENK-WT*^ littermates. Sholl analysis further supported this observation, revealing no difference in dendritic arbor complexity (Fig. 3d). Although these morphological data apply to the total MSN population, there was no evidence for a bimodal distribution, making unlikely the possibility that defects in ENK^+^MSNs were masked by the striatonigral subpopulation. However, striatal enkephalin expression was reduced in *Trkb*^*PENK-KO*^ mice in the absence of striatal MSN loss or DARPP-32 reduction (Fig. 3b, and Supplementary Fig. S6e). Further analysis of synaptic markers and dopamine receptors levels in the striatum revealed no significant changes between mutants and controls at 12 M of age (Fig. 3e). These results demonstrate that long-term survival and morphology of striatopallidal MSNs are not affected by BDNF–TrkB signaling-specific inactivation. In contrast, depletion of TrkB signaling affects striatal enkephalin expression and function of these neurons, suggesting that signaling activated by BDNF–TrkB is required in these cells to perform normal inhibitory function on locomotor activity.

### Intact corticostriatal synaptic properties in *Trkb*^*PENK-KO*^ mice

**Figure 4:**
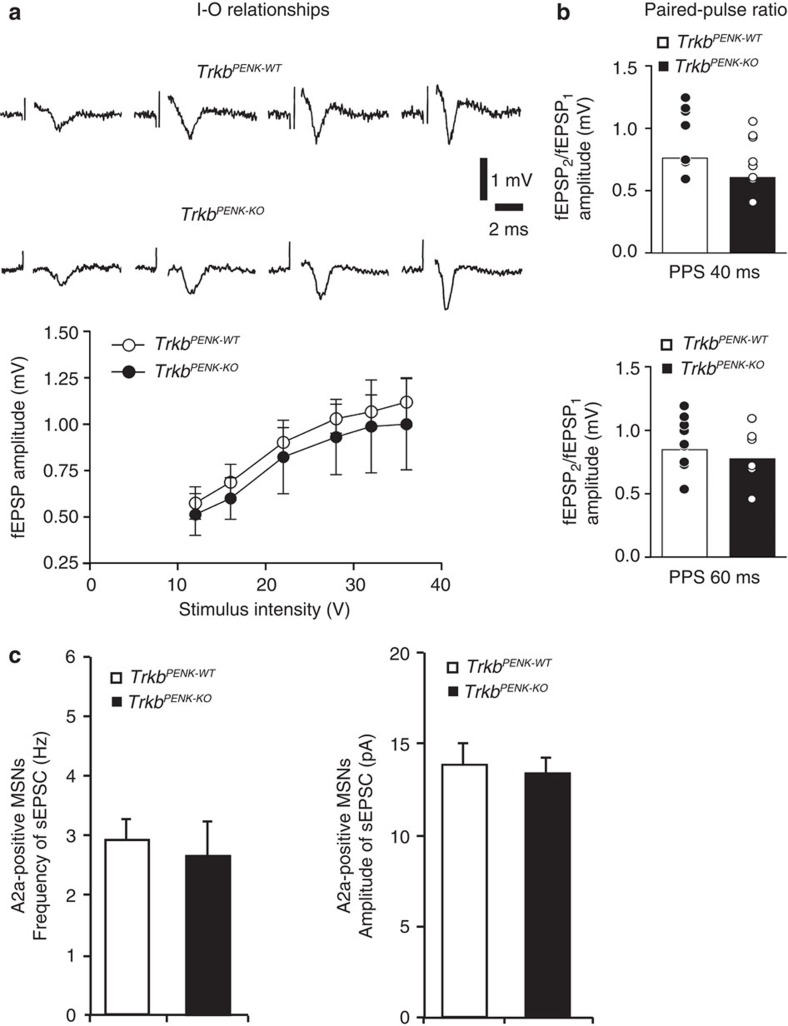
Functional integrity of the corticostriatal pathway in *Trkb*^*PENK-KO*^ mice. (**a**) Averaged fEPSP traces (upper panel) evoked by a stimulation of 12, 16, 22, 28, 32 and 36 V. Input–output best-fit curves (lower panel) revealed no significant difference between *Trkb*^*PENK-WT*^ (*n*=7 slices/4 mice) and mutant mice (*n*=10 slices/4 mice) (*P*>0.05, unpaired Student’s *t*-test). Values are means±s.e.m. of fEPSP amplitude evoked by a given stimulation. (**b**) Histograms of group data showing similar paired-pulse ratio (PPR) of responses at 40 and 60 ms of interpulse intervals stimulation between *Trkb*^*PENK-WT*^ (*n*=8 slices/4 mice) and *Trkb*^*PENK-KO*^ mice (*n*=9 slices/4 mice), and similar ratio values (*P*>0.05, unpaired Student’s *t*-test). Values are means±s.e.m. of changes in the respective cell populations. (**c**) Graphs showing mean frequency and amplitude of the spontaneous postsynaptic currents (sEPSC) recorded from A2A-positive MSNs (*Trkb*^*PENK-KO*^, frequency: 2.67±0.56 (*n*=9/5 mice) versus *Trkb*^*PENK-WT*^: 2.94±0.32 (*n*=14/9 mice), *P*=0.64, unpaired Student’s *t*-test; *Trkb*^*PENK-KO*^, amplitude: 13.3±0.87 (*n*=9/5 mice) versus *Trkb*^*PENK-WT*^: 13.8±1.2 (*n*=14/9 mice), *P*=0.76, unpaired Student’s *t*-test. Values are means±s.e.m.

As alterations of the corticostriatal tract are known to affect MSN physiology and, therefore, locomotor behavior, we examined the electrophysiological properties of this tract in *Trkb*^*PENK-WT*^ and *Trkb*^*PENK-KO*^ mice. Basal glutamatergic synaptic transmission in slices containing the dorsolateral striatum of *Trkb*^*PENK-WT*^ and *Trkb*^*PENK-KO*^ mice was analyzed by extracellular field potential recordings. Input–output relationships were constructed by plotting the mean amplitude of field excitatory postsynaptic potential (fEPSP) against the increasing stimulation intensities. There was no significant difference in the input–output curves or in the amplitude of the fEPSPs between *Trkb*^*PENK-WT*^ and mutant mice (Fig. 4a). To determine whether ablation of *Trkb* from striatopallidal neurons would affect neurotransmitter release from presynaptic terminals at corticostriatal synapses, we measured responses to paired-pulse stimulation at 40 and 60 ms interpulse intervals, whose ratio is considered as an index of release probability, a form of short-term synaptic plasticity. Paired-pulse responses had similar amplitude between *Trkb*^*PENK-WT*^ and *Trkb*^*PENK-KO*^ mice, and similar ratio values (Fig. 4b). We also examined another crucial parameter of basal glutamatergic transmission, namely, the spontaneous activity of MSNs. Interestingly, no significant differences in frequency or amplitude of the spontaneous postsynaptic currents were found in ENK^+^MSNs between controls and mutants (Fig. 4c). Collectively, these findings indicate that basal excitatory synaptic transmission and short-term plasticity at the corticostriatal synapses are similar in the two genotypes. However, these data do not rule out the possibility that lack of BDNF/TrkB signaling in striatopallidal neurons would lead to other electrophysiological changes.

### TrkB signaling modulates striatopallidal MSN activation

**Figure 5:**
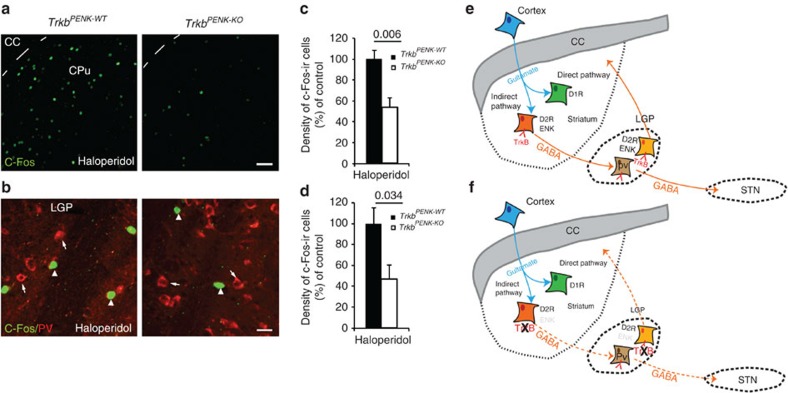
Reduced neuronal activation in *Trkb*-depleted striatopallidal cells in response to D2R-blockade. (**a**–**b**) Representative images of CPu (**a**) and LGP (**b**) showing c-Fos expression 2 h after systemic i.p. injection of 1 mg kg^−1^ haloperidol in 3-month-old *Trkb*^*PENK-WT*^ and *Trkb*^*PENK-KO*^ mice (*n*=5, each genotype). In (**b**) tissues are co-labeled with an Ab against parvalbumin. These cells are the target of the striatopallidal MSNs and do not express D2R/ENK. Therefore, these cells are insensitive to haloperidol in contrast to the other cell type within the LGP that is D2R/ENK+ (PV−)^32^. (**c,d**) Quantification of density of haloperidol-induced c-Fos-ir cells in the CPu (**c**) (*Trkb*^*PENK-WT*^, 100±8.81 *n*=5, *Trkb*^*PENK-KO*^, 53.03±9.40 *n*=5, *P*=0.006, unpaired Student’s *t*-test), and LGP (**d**) (*Trkb*^*PENK-WT*^, 100±15.86 *n*=5, *Trkb*^*PENK-KO*^, 46.52±13.68 *n*=5, *P*=0.034, unpaired Student’s *t*-test); values are means±s.d. (**e,f**) Schematic representation of TrkB signaling requirement in striatopallidal MSNs, and relative neuronal populations in the CPu and the LGP. Red and green cells in the dorsal striatum represent respectively striatopallidal (indirect pathway) and striatonigral (direct pathway) MSNs. Brown and orange cells in the LGP represent respectively PV+ neurons (indirect pathway) that projects to the subthalamic nucleus (STN), and PV−, D2R/ENK+ pallidostriatal neurons that project to the striatum^32^. (**e**) Glutamatergic input from the cortex to the striatopallidal MSNs (D2R/ENK+) is integrated in these neurons by functional dopamine/D2R and BDNF–TrkB activation. This leads to normal transmission of inhibitory signal (GABA) to the target in the LGP, PV+ neurons. (**f**) Depletion of TrkB signaling alters indirect pathway MSN activation causing diminished inhibitory signaling to the target cells in the LGP (PV+), and PV−, D2R/ENK+ pallidostriatal neurons causing impaired feedback to the striatum. CC, corpus callosum; CPu, caudate-putamen; LGP, lateral globus pallidum; PV, parvalbumin. SNT, subthalamic nucleus. Arrowheads and arrows indicate c-Fos positive and PV-positive cells, respectively. Scale bars: **a**, 50 μm; **b**, 20 μm. See also Supplementary Methods.

As *Trkb* deletion in ENK^+^MSNs results in spontaneous and drug-induced hyperlocomotion, we next asked if TrkB signaling in these neurons would interfere with dopamine/D2R function that represses striatopallidal MSNs excitability in response to glutamatergic cortical input. Therefore, we combined precise mouse genetics with a pharmacological approach, and determined whether loss of *Trkb* specifically from D2R/ENK-positive MSNs would impair the response of these cells upon acute excitatory effect induced by D2R blockade. We performed D2 receptor blockade using a D2R-like antagonist (haloperidol)^31^, which triggers an acute excitatory effect of striatopallidal MSNs. This is followed by neuronal activation and c-Fos induction predominantly in D2R neurons^31^. Accordingly, *Trkb*^*PENK-WT*^ and *Trkb*^*PENK-KO*^ animals were injected either with 1 mg kg^−1^ haloperidol or vehicle (DMSO), and the density of c-Fos-immunoreactive (c-Fos-ir) cells in the caudate-putamen was determined after 2 h by immunohistochemistry. The results showed a significant reduction in the density of haloperidol-induced c-Fos-ir cells in *Trkb*^*PENK-KO*^ mice compared with that in *Trkb*^*PENK-WT*^ (Fig. 5a). These data indicate that *Trkb*-depleted striatopallidal MSNs are not able to respond efficiently to the excitatory effect induced by D2R blockade, suggesting that TrkB signaling modulates activation of striatopallidal MSNs integrating cortical glutamatergic input.

To further support the observations obtained in the dorsal striatum, we also assessed the ability of haloperidol to activate D2R/ENK+(PV-) MSNs in the LGP of *Trkb*^*PENK-KO*^ mice and *Trkb*^*PENK-WT*^ by similar analysis of c-Fos-ir expression in response to D2R blockade. These cells project to the striatum and form the pallidostriatal pathway^32^. The results obtained 2 h after haloperidol injection show a significant reduction in the density of haloperidol-induced c-Fos-ir cells in *Trkb*^*PENK-KO*^ mice compared with that in *Trkb*^*PENK-WT*^ (Fig. 5b). These data indicate impaired feedback of PV-negative LGP cells to striatal neurons. This effect was similar to that observed in the striatopallidal MSNs of the indirect pathway, and indicates that TrkB signaling is required in these cells for their normal activation.

### Intact striatopallidal projections in the absence of TrkB

**Figure 6:**
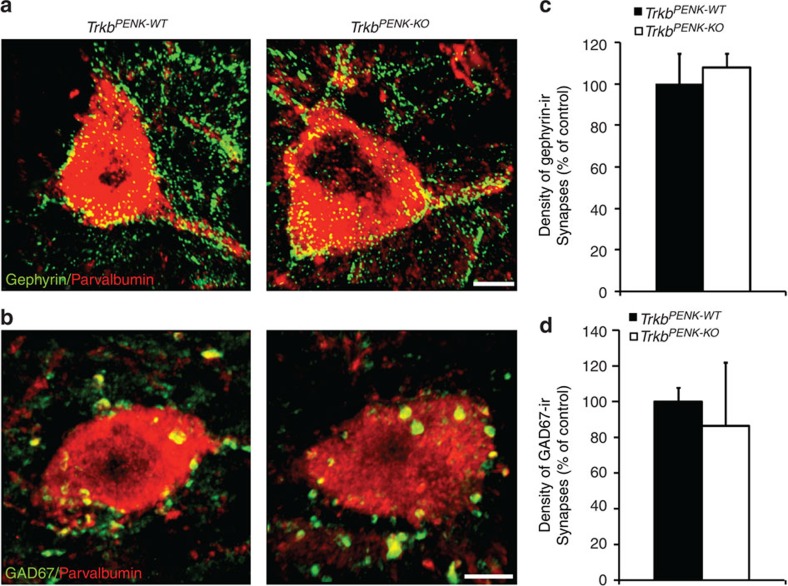
Unchanged synaptic density in striatopallidal targets in the absence of presynaptic BDNF–TrkB signaling. (**a,b**) Single-cell level confocal images of LGP neurons co-labeled with parvalbumin (red) and gephyrin (green) (**a**), or parvalbumin (red) and GAD67 (green) (**b**), in 3-M-old haloperidol-treated *Trkb*^*PENK-WT*^ and *Trkb*^*PENK-KO*^ mice highlighting somatodendritic synapses. (**c,d**) Density of GABAergic synapses in pallidostriatal neurons quantified by gephyrin (*Trkb*^*PENK-WT*^, 100±14.59 *n*=20 neurons from 3 mice, *Trkb*^*PENK-KO*^, 107.66±7.04 *n*=19 neurons from three mice, *P*=0.46, unpaired Student’s *t*-test) (**c**); and GAD67 (*Trkb*^*PENK-WT*^, 100±7.78, *n*=27 neurons from three mice, *Trkb*^*PENK-KO*^ 86.38±35.79, *n*=24 neurons from three mice, *P*=0.55, unpaired Student’s *t*-test) (**d**). Values are means±s.d. Scale bars: a–b, 5 μm.

To analyze if reduced activation of the striatopallidal neurons would affect targeting of striatopallidal projections in the LGP, we analyzed the density of GABAergic synapses on PV+ LGP neurons, which are part of the indirect pathway and receive direct striatopallidal projections^33^. Densities of pre- and postsynaptic GABAergic components were analyzed by immunostaining of the presynaptic enzymes glutamic acid decarboxylase 67 (GAD67) and the postsynaptic scaffolding protein gephyrin, respectively. The imaging at single-cell level of PV-expressing pallidal cells paired with 3D computer-based reconstruction of the immunostaining allowed detailed analysis of the GABAergic striatopallidal terminations. Quantification of either gephyrin- or GAD67-containing synapses on PV-labeled neurons in the LGP of haloperidol-treated *Trkb*^*PENK-WT*^ and *Trkb*^*PENK-KO*^ mice revealed no significant difference in the somatodendritic density of both GAD67 and gephyrin (Fig. 6a–d). These data indicate that absence of TrkB signaling does not affect targeting of striatopallidal projections and/or synaptic density on LGP PV+ neurons.

### TrkB modulates D2R-dependent striatal PKC and MAPK activation

**Figure 7:**
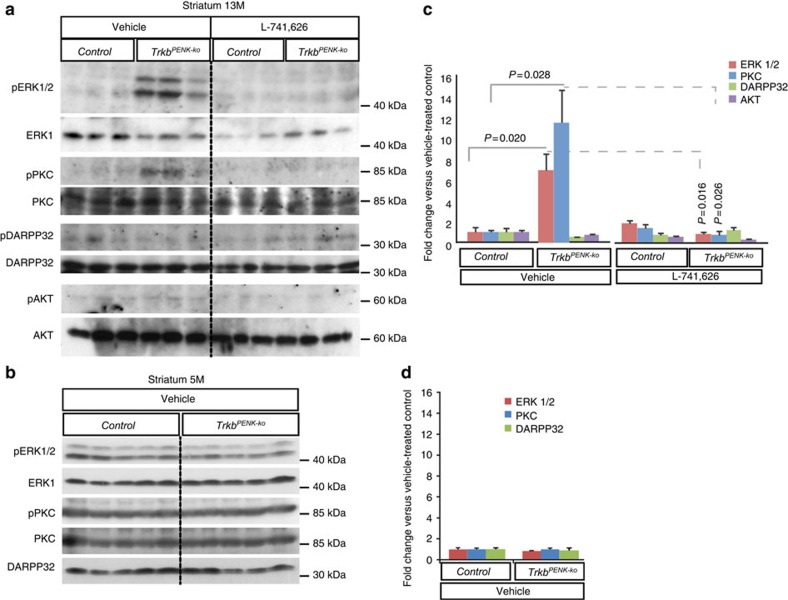
ENK-specific *Trkb* deletion affects striatal D2R-dependent PKC and ERK1/2 phosphorylation. (**a,b**) Representative immunoblots of proteins extracted from dorsal striatum of 13-M- and 5-M-old *Trkb*^*PENK-WT*^ and *Trkb*^*PENK-KO*^ mice. Animals were either treated with vehicle or with a D2R-specific antagonist L-741,626 (20 mg/kg), and killed 30 min later. Phosphorylation states of ERK1/2, PKC, AKT and DARPP-32 were determined using phospho-specific Abs. See also Supplementary Fig. S8b–c. (**c**) Graphs represent the phosphorylation state of each protein normalized to its total amount. Fold changes were determined against vehicle-treated *Trkb*^*PENK-WT*^ (set as 1), (vehicle: *Trkb*^*PENK-KO*^ versus *Trkb*^*PENK-WT*^, pERK1/2, *P*=0.020; p-PKC, *P*=0.028; p-DARPP32, *P*=0.065; pAKT, *P*=0.17), (L-741,626: *Trkb*^*PENK-KO*^ versus *Trkb*^*PENK-WT*^, pERK1/2, *P*=0.07; p-PKC, *P*=0.19), (L-741,626 treated *Trkb*^*PENK-KO*^ versus vehicle treated *Trkb*^*PENK-KO*^, pERK1/2, *P*=0.016; p-PKC, *P*=0.026). *P*-values were generated by unpaired Student’s *t*-test. Values are means±s.e.m., *n*=6 per genotype (three per condition) analyzed. (**d**) Graphs represent the phosphorylation state of each protein normalized to its total amount at 5 M of age. Total DARPP-32 was normalized to ERK1. Fold changes were determined against vehicle-treated *Trkb*^*PENK-WT*^ (set as 1), (vehicle: *Trkb*^*PENK-KO*^ versus *Trkb*^*PENK-WT*^, pERK1/2, *P*=0.06; p-PKC, *P*=0.90; DARPP-32, *P*=0.33). *P*-values were generated by unpaired Student’s *t*-test. Values are means±s.e.m., *n*=5 per group analyzed.

It has been shown that D2 receptor agonists lead to MAPK phosphorylation in neurons through increased intracellular Ca^2+^ and PKC activity^34^. Striatal activation of ERK is necessary for the expression of D2 receptor-mediated locomotor hyperactivity^35^. Therefore, to provide a possible mechanistic insight into our novel findings, we have tested the effect of TrkB signaling depletion on the activation of these kinases in 13-M-old *Trkb*^*PENK-KO*^ striatum, when mutants already show spontaneous hyperlocomotion, and in 5-M-old, a presymptomatic stage. We found significantly enhanced phosphorylation of both PKC and ERK1/2 in *Trkb*^*PENK-KO*^ mice compared with that in *Trkb*^*PENK-WT*^ at 13 M of age, but no significant difference in AKT or DARPP-32 (Thr34) phosphorylation (Fig. 7a). The enhanced phosphorylation of PKC and MAPKs was not evident in *Trkb*^*PENK-KO*^ mice at 5 M of age, suggesting that aging is a critical factor (Fig. 7b). To test whether the increased phosphorylation state of PKC and ERK1/2 was D2R-dependent, we challenged 13-M-old *Trkb*^*PENK-KO*^ mice and *Trkb*^*PENK-WT*^ with a more selective antagonist of the D2R (L-741,626) (ref. 36). This treatment blocked the increased phosphorylation of PKC and ERK1/2 in *Trkb*^*PENK-KO*^ mice (Fig. 7a), indicating that increased PKC/MAPK phosphorylation is the result of enhanced D2R-signaling in the absence of TrkB signaling.

## Discussion

The specific depletion of TrkB signaling from mouse striatopallidal neurons, while maintaining cerebral cortex TrkB expression, has allowed to investigate the physiological role of BDNF–TrkB signaling in ENK^+^MSNs and to determine whether the known effects of altered cortical BDNF signaling on striatal vulnerability are mediated via TrkB. Here, we show that TrkB signaling is required to control inhibition of locomotor behavior in ENK^+^MSNs. Ablation of *Trkb* from striatopallidal MSNs results in spontaneous and drug-induced hyperlocomotion associated with reduced enkephalin expression and increased D2R-dependent striatal PKC and MAPKs phosphorylation, leading to altered response of ENK^+^MSNs to glutamate-mediated excitation. However, long-term survival and morphology of ENK^+^MSNs were not affected. Finally, these data identify a critical role for TrkB signaling in locomotor behavior exerted by ENK striatopallidal neurons and demonstrate that these neurons are not dependent on BDNF–TrkB signaling for long-term survival and morphology *in vivo*.

BDNF is a major regulator of neuronal survival and morphology. One current main hypothesis suggests that a reduction in cortical BDNF and/or impairment in BDNF anterograde transport from cortex to striatal targets contribute to the degeneration of striatal neurons observed both in patients and in HD animal models^37^. This suggestion, however, is based mainly on genetic models that compromise cerebral cortex physiology, and do not allow direct evaluation of the contribution of BDNF–TrkB signaling to striatal vulnerability. Therefore, to address the physiological role of BDNF–TrkB signaling in striatopallidal neurons, the most vulnerable neuronal subtype in HD^3^, we have selectively deleted the high-affinity BDNF receptor, TrkB, from these neurons. Our data indicate that BDNF–TrkB signaling is required to control inhibition of locomotor behavior, but is not an essential factor for long-term survival and maintenance of ENK MSNs, and does not have a direct role in the striatal vulnerability observed upon cortical deletion of *Bdnf*. These contradictory roles for BDNF may be explained by its necessity for the maintenance of cortical neuron morphology. In particular, forebrain-specific conditional deletion of *Bdnf* leads to a reduction in cellular size and dendritic complexity of pyramidal neurons in cortical layer II–III at just 3 weeks of age^38^. This, and possible alteration of other cortical layers, could compromise cortical input to striatal targets leading to the observed morphological changes in striatal MSNs^14^. Therefore, in contrast to the current belief^14^,^39^, our data support the theory that complex alteration of the corticostriatal tract, and/or cortical cell-intrinsic properties ultimately contribute to striatal vulnerability. Indeed, dysfunction of the corticostriatal tract seems to occur during the development of the HD phenotype before any significant neuronal cell loss is apparent in the striatum^40^,^41^,^42^,^43^. In our mouse model, we found functional integrity of the corticostriatal tract analyzed by electrophysiology. The procedure we have used (fEPSP) has been shown to be sensitive and reliable for detecting alterations in the electrophysiological properties of MSN subpopulations^44^.

Unexpectedly, disruption of TrkB signaling in ENK^+^MSNs induces spontaneous hyperlocomotion, suggesting that TrkB in striatopallidal neurons contributes to the inhibitory control of locomotor behavior exerted by the indirect pathway. Notably ablation of D2R neurons in the entire striatum produced a bigger hyperlocomotor response than when ablation was limited to ventral striatum only^25^. This is in agreement with the current hypothesis of the BG identifying striatopallidal MSNs as an inhibitory component of the circuit controlling motor activity^45^. Particularly noteworthy is that conditional inactivation of TrkB signaling in striatopallidal MSNs leads to a reduction of enkephalin, one of the earliest morphological changes observed in HD^3^,^46^, without affecting MSN viability. The decrease in enkephalin expression presumably reflects a reduction in neuronal activity of the striatopallidal efferents projecting to the external globus pallidus. This could affect the identity of MSN belonging to the indirect pathway, compromising their functionality and consequently causing an increase of spontaneous and cocaine-associated activity by disinhibition of the striatopallidal projections. Indeed, a similar phenotype occurs by conditional deletion of DARPP-32 in striatopallidal MSNs. This leads to increased basal and cocaine-induced locomotor activity in the presence of a compromised corticostriatal LTP without any cell loss^26^. In our model, however, since cortical *Trkb* has been left mainly intact, the functional integrity of the corticostriatal pathway is not affected and the inhibitory function observed is therefore mainly confined to the striatopallidal pathway. Recent studies though, showed that mice carrying deletion of *Trkb* mediated by a less-restricted Cre line (D_2_R-Cre)^47^, have decreased locomotor activity upon cocaine administration without significant changes of c-Fos-ir in the dorsal striatum upon acute cocaine treatment^48^. This inconsistency with our study may be due to D_2_R expression in both striatopallidal MSNs and striatal cholinergic interneurons^47^, unlike enkephalin. Therefore, considering the modulatory activity of striatal cholinergic cells over the striatopallidal pathway^49^,^50^, it is not surprising that the opposite result was obtained in the activation of striatopallidal neurons and, therefore, in the behavioral output.

It is evident from the classical BG model^10^ that normal locomotor behavior results from the balance between direct and indirect BG pathways. Recently, this model has also been supported by the use of optogenetics^51^. Moreover, D2R activation reduces indirect pathway excitability specifically induced by cortical afferent activation and subsequent Ca^2+^ inflow^52^. Diminished D2R or a D2R blockade leads to an excess of Ca^2+^ inflow, hence increasing indirect pathway activation. Essentially, D2R activation represses striatopallidal MSNs excitability by inhibiting calcium entry in these neurons through modulation of NMDAR and R-type Ca^2+^ channels. This effect is, in part, compensated by A2AR activation, which enhances Ca^2+^ entry^52^. In our model, striatopallidal MSNs carrying TrkB depletion are not able to respond efficiently to the acute excitatory effect induced by D2R-like antagonist haloperidol (measured by c-Fos induction). The observed reduced enkephalin expression in these neurons also supports this finding. This suggests that TrkB signaling modulates activation of striatopallidal MSNs, integrating cortical glutamatergic input, most likely by enhancing Ca^2+^ entry to compensate for the dopamine/D2R repression of glutamatergic input, therefore maintaining physiological balance of GABAergic transmission. In addition, we found that major manifestation of phenotypes, such as hyperlocomotion, is noticeable with age or by challenges such as cocaine administration in young mutant animals. One possible explanation for this is that during aging there is a progressive decline in brain production of neurotrophic factors and their cognate receptors. In the BG of young animals, the presence of a significant amount of trophic factors may be able to compensate for the deficit caused by the absence of TrkB signaling. With aging, the availability of trophic factors declines, exacerbating the deficit caused by the absence of TrkB. Epigenetic changes occurring with aging could also explain the appearance of the phenotype only in older animals.

Finally, *Trkb*-specific deletion in ENK^+^MSNs demonstrates that in physiological conditions, BDNF signaling is not required for the long-term survival and morphology of these striatal neurons. It does, however, affect enkephalin expression and neuronal activity resulting in altered locomotion. As reduced levels of BDNF in a diseased brain exacerbate the severity of motor dysfunction^5^, its replacement should maintain normal cortical physiology, and, therefore, also ameliorate striatal physiology by re-establishing enkephalin levels and neuronal activity, and thus improve motor behavior^8^. Our data do not exclude the possibility that the concomitant absence of BDNF–TrkB signaling in the presence of mutant huntingtin in striatal MSNs could instead have a deleterious effect on striatal neuron physiology. These findings have obvious relevance given potential therapeutic approaches to HD.

## Methods

### Ethics statement

All animal procedures at the European Molecular Biology Laboratory (Mouse Biology Unit) conformed to National and International laws and policies (EEC Council Directive 86/609, OJ L 358, 1, 12 December 1987; NIH Guide for the Care and Use of Laboratory Animals, NIH Publication No. 85-23, 1985, revised in 1995); at the Centre for Neuroregeneration and at the Department of Pharmacology, animal procedures conformed to UK legislation (Scientific procedures) ACT 1986, the University of Edinburgh and the University of Oxford ethical review committee policy, respectively.

### Immunohistochemistry

Immunohistochemistry was performed as previously described^23^. Primary Abs were used at the following dilution: Ctip2 (1:1,000, ab18465, Abcam), DARPP-32 (1:500, AB1656, Chemicon), EGFP (1:500, ab290, Abcam), c-Fos (1:500, sc-52, Santa Cruz Biotechnology), enkephalin (1:200, RA14124, Neuromics), ChAt (1:1,000, ab1449, Chemicon), Calretinin (1:1,000, 7699/4, Swant), Somatostatin (1:200, ab5494, Chemicon), Parvalbumin (1:1,000, P3088, Sigma, rabbit polyclonal), GAD67 (1:2,000, Millipore) and gephyrin (1:500, Synaptic System). Alexa-labeled secondary Abs (1:1,000 Invitrogen), or biotin-conjugated anti-rabbit Ab (1:500 Jackson Lab). All fluorescent images were taken on LSM710 Meta confocal microscope (Carl Zeiss) mounting 40x/0.75 NA, and × 63/1.4 NA planapochromat objectives (Carl Zeiss). Bright field images were taken using Axio Scope (Carl Zeiss) mounting × 20/0.8 NA and × 40/0.75 NA planapochromat objectives (Carl Zeiss), coupled with an Axio Cam ICC1 color camera (Carl Zeiss).

### Striatal cell counts

The total number of striatal MSNs defined by fluorescent DARPP-32 expression and *in situ* labeled enkephalin-expressing MSNs (Supplementary Methods) were estimated using design-based stereology with the optical fractionator method as described by Gundersen *et al*.^53^ Sequential sagittal sections with a thickness of 30 μm spanning the entire medio-lateral extension of one brain hemisphere were obtained at the cryostat (Leica Microsystems). One in every 12 sections were counted with an unbiased counting frame size 50 × 30 μm for DARPP-32 and 100 × 100 μm for *Enk*. The frames were randomly positioned by the ‘Unbiased counting frame’ macro of ImageJ (v1.46r, NIH) with a regular distance between frames of *x*=300 μm, *y*=280 μm for DARPP-32 and *x*=330 μm, *y*=300 μm for *Enk*. The heights of the dissectors were of 8 and 15 μm with a guard of 4 and 2 μm on either side, respectively (Supplementary Fig. 7). Cells in each frame were counted complying with the optical fractionator counting rules. Striatal boundaries were defined using the following morphological references: the corpus callosum, the external capsule, the lateral ventricle and the anterior commissure.

### Morphological analysis of MSNs including spine density

The morphological analysis of striatal neurons was based on previously described Golgi–Cox impregnation method reported to be highly sensitive for detecting morphological alteration of striatal cells^8^. More details are reported in Supplementary Methods.

### Haloperidol treatment

Three-month-old *Trkb*^*PENK-KO*^ and *Trkb*^*PENK-WT*^ mice were intraperitoneally (i.p.) injected with 1 mg kg^−1^ haloperidol (H1512, Sigma) or vehicle (DMSO), and 2 h later, were euthanatized, perfused transcardially and processed for immunohistochemistry as described above.

### L-741,626 treatment

13-M-old mutants and controls were either i.p.-injected with 20 mg kg^−1^ L-741,626 or with vehicle (DMSO), and 30 min later, animals were killed by cervical dislocation and striata were dissected, followed by protein lysate preparation and immunoblots, as described below.

### Immunoblotting

Protein lysates from adult controls and mutant mouse brains were obtained by dissection of the cortex and striatum. Tissues were homogenized in 10 volumes of (vol/wt) lysis buffer (150 mM NaCl, 50 mM Tris pH 8.0, 1%(vol/vol) NP-40, 0.1%(wt/vol) SDS plus a cocktail of protease inhibitors, Sigma) by a glass dounce (VWR), followed by mild sonication. Total protein lysate (30 μg) were then subjected to SDS–PAGE. After transfer onto nitrocellulose membranes (GE healthcare), membranes were probed with Ab against TrkB (1:500, 80E3, Cell Signaling), Synaptophysin (1:10,000, Sy-Sy), PSD-95 (1:1,000, Millipore), Neuroligin-2 (1:500, Sy-Sy), GAD67 (1:1,000, Millipore), DARPP-32 (1:2,000, Millipore), D2R (1:200, Millipore), D1R (1:200, Millipore), ERK (1:1,000, Zymed), PKC_consensus_ (1:1,000, Calbiochem), AKT (1:1,000, Cell Signaling), GAPDH (1:10,000, Sigma) or TUJ.1 (1:10,000, Abcam); for the detection of phosphorylated proteins, membranes were incubated with Abs from Cell Signaling against p-PKC (pan, Thr514), pMAPK (Erk1/2) (Thr202/Tyr204), p-DARPP-32 (Thr34), p-AKT (Ser473), followed by incubation with HRP-conjugated secondary Ab (1:10,000, Jackson Lab). Images were visualized by using SuperSignal West Pico Chemiluminescent Substrate System (Pierce) and BioMax Light Films (Kodak).

### Behavioral analysis

The body weights of mice involved in the behavioral tests were noted before every task.

OF: spontaneous locomotor activity was assessed by analyzing exploratory behavior of 2–14-month-old mice of both sexes in a 50 × 50 × 28 cm open arena (TSE, Germany) illuminated with 100 lux and coupled with an automated video tracking system (VideoMot, TSE). Each mouse was placed in the center of the field, and allowed to explore the arena freely for 30 min. For the experiments addressing cocaine-induced locomotion, mice were weighed before every session in order to determine the amount of drug to deliver. Animals on day −1 were placed in the arena and left to habituate to the apparatus for 30 min; on day 0, mice were subjected to a second habituation session immediately after i.p. vehicle injection (NaCl, 0.9% wt/vol). In the subsequent 3 days, cocaine-paired sessions were performed after i.p. injection of 10 mg kg^−1^ of cocaine hydrochloride (Sigma) dissolved in 0.9% NaCl solution, following the same procedure. The time spent in the center (arbitrarily defined as 34 × 34 cm square inside the OF) of the arena and the total distance traveled were measured. The average across the 3 cocaine-conditioned days is reported.

The accelerating rotarod system was used to assess motor coordination (Advanced System, TSE). On day 1, 2–14-month-old mice of both sexes were habituated to the apparatus by subjecting them to three sessions of 3 min at 4 r.p.m. constant speed, separated by 15-min rest intervals. The next-day performance was tested by placing mice on the rod accelerating from 4 to 40 r.p.m. (over a 5-min interval). Mice were scored for the latency to fall during the trials. The average latency of the three trials for each animal was then calculated.

For the inverted screen test, 5-month--old mice of both sexes were placed in the center of a screen (20.5 × 13 cm-wire mesh, 12 mm^2^ holes), and the screen was inverted over a 2-s period with the mouse’s head declining. The screen was held steadily 90 cm above a solid, cushioned surface. Latency to fall was noted with cutoff time at 90 s.

For the weight-lifting/grip strength test, 5-month-old mice of both sexes were held by the tail and lowered to grasp a scale collector (a ball of tangled fine stainless steel wire, 7 g) attached to increasing lengths of steel chain made up of 13 g links. Each mouse was then raised for 3 s, supporting the weight with its forepaws. Mice were allowed to rest for 5 min before progressing to the next heavier weight. If the mouse dropped the weight immediately, it was tested again 10 s later. Failure at the second trial terminated the test. Maximum weight supported by the mouse for 3 s was used as a measure of strength.

Detailed analysis of gait was performed using the CatWalk system (Noldus, the Netherlands) according to the previously published method^28^,^29^,^54^. Runs were recorded using a camera (GEViCAM Inc., USA). Details of the general parameters measured and analyzed are reported in Supplementary Methods.

### Electrophysiological experiments

The electrophysiological recordings were performed according to Calabresi *et al*.^55^ Adult male mice (5–9 months) were used for all the experiments. Details are reported in the Supplementary Methods.

### Statistical analysis

Two-way ANOVA using either age and genotype, or dendritic order and genotype as independent variables, was used for the data analysis of the rotarod, and spontaneous locomotion or spine density and dendritic thickness, respectively. One-way ANOVA was used to determinate significance between different genotypes in Sholl analysis and for cocaine-induced locomotion analysis. In case of significant difference among the means, a *post hoc* Fisher PLSD test was performed and the *P*-value was reported. Cell counts, synaptic density, WB analysis, locomotion of *BAC–PENK*^*tg/+*^, electrophysiological data, and behavioral data for the CatWalk, inverted screen and grip strength were analyzed by two-tailed unpaired Student’s *t*-test. All tests of significance were performed at *α*=0.05 using Unistat 5.6 (Unistat Ltd, London, UK).

## Author contributions

D.B. planned and performed most of the experiments. M.G. conducted the *in situ* experiments. D.K. contributed to the characterization of the transgenic BAC–Cre line used in this study. B.P., V.P. and P.C. provided the expertise for and conducted the electrophysiological experiments. T.S. and D.M.B. provided the expertise for and conducted the fine motor behavioral analysis. L.M. is the principal investigator and contributed to the experimental plans, supervised the project, provided theoretical input and wrote the manuscript.

## Additional information

**How to cite this article:** Besusso, D. *et al*. BDNF–TrkB signaling in striatopallidal neurons controls inhibition of locomotor behavior. *Nat. Commun.* 4:2031 doi: 10.1038/ncomms3031 (2013).

## Supplementary Material

Supplementary Information Supplementary Figures S1-S8, Supplementary Tables S1-S3, Supplementary Methods and Supplementary References

## References

[b1] Calabresi P., Picconi B., Tozzi A. & Di Filippo M. Dopamine-mediated regulation of corticostriatal synaptic plasticity. Trends Neurosci. 30, 211–219 (2007). 17367873 10.1016/j.tins.2007.03.001

[b2] Kreitzer A. C. & Malenka R. C. Striatal plasticity and basal ganglia circuit function. Neuron 60, 543–554 (2008). 19038213 10.1016/j.neuron.2008.11.005 PMC2724179

[b3] Reiner A., Albin R. L., Anderson K. D., D’Amato C. J., Penney J. B. & Young A. B. Differential loss of striatal projection neurons in Huntington disease. Proc. Natl Acad. Sci. USA 85, 5733–5737 (1988). 2456581 10.1073/pnas.85.15.5733 PMC281835

[b4] Andrew S. E. . The relationship between trinucleotide (CAG) repeat length and clinical features of Huntington's disease. Nat. Genet. 4, 398–403 (1993). 8401589 10.1038/ng0893-398

[b5] Canals J. M. . Brain-derived neurotrophic factor regulates the onset and severity of motor dysfunction associated with enkephalinergic neuronal degeneration in Huntington’s disease. J. Neurosci. 24, 7727–7739 (2004). 15342740 10.1523/JNEUROSCI.1197-04.2004 PMC6729627

[b6] Sapp E. . Evidence for a preferential loss of enkephalin immunoreactivity in the external globus pallidus in low grade Huntington's disease using high resolution image analysis. Neuroscience 64, 397–404 (1995). 7535402 10.1016/0306-4522(94)00427-7

[b7] Richfield E. K., Maguire-Zeiss K. A., Cox C., Gilmore J. & Voorn P. Reduced expression of preproenkephalin in striatal neurons from Huntington's disease patients. Ann. Neurol. 37, 335–343 (1995). 7695232 10.1002/ana.410370309

[b8] Xie Y., Hayden M. R. & Xu B. BDNF overexpression in the forebrain rescues Huntington's disease phenotypes in YAC128 mice. J. Neurosci 30, 14708–14718 (2010). 21048129 10.1523/JNEUROSCI.1637-10.2010 PMC2989389

[b9] Graybiel A. M. The basal ganglia. Curr. Biol. 10, R509–R511 (2000). 10899013 10.1016/s0960-9822(00)00593-5

[b10] Albin R. L., Young A. B. & Penney J. B. The functional anatomy of basal ganglia disorders. Trends Neurosci. 12, 366–375 (1989). 2479133 10.1016/0166-2236(89)90074-x

[b11] Zuccato C. & Cattaneo E. Role of brain-derived neurotrophic factor in Huntington's disease. Prog. Neurobiol. 81, 294–330 (2007). 17379385 10.1016/j.pneurobio.2007.01.003

[b12] Altar C. A. . Anterograde transport of brain-derived neurotrophic factor and its role in the brain. Nature 389, 856–860 (1997). 9349818 10.1038/39885

[b13] Gauthier L. R. . Huntingtin controls neurotrophic support and survival of neurons by enhancing BDNF vesicular transport along microtubules. Cell 118, 127–138 (2004). 15242649 10.1016/j.cell.2004.06.018

[b14] Baquet Z. C., Gorski J. A. & Jones K. R. Early striatal dendrite deficits followed by neuron loss with advanced age in the absence of anterograde cortical brain-derived neurotrophic factor. J. Neurosci. 24, 4250–4258 (2004). 15115821 10.1523/JNEUROSCI.3920-03.2004 PMC6729276

[b15] Strand A. D. . Expression profiling of Huntington's disease models suggests that brain-derived neurotrophic factor depletion plays a major role in striatal degeneration. J. Neurosci. 27, 11758–11768 (2007). 17959817 10.1523/JNEUROSCI.2461-07.2007 PMC6673215

[b16] Ferrer I., Goutan E., Marín C., Rey M. J. & Ribalta T. Brain-derived neurotrophic factor in Huntington disease. Brain Res. 866, 257–261 (2000). 10825501 10.1016/s0006-8993(00)02237-x

[b17] Kuhn A. . Mutant huntingtin's effects on striatal gene expression in mice recapitulate changes observed in human Huntington's disease brain and do not differ with mutant huntingtin length or wild-type huntingtin dosage. Hum. Mol. Genet. 16, 1845–1861 (2007). 17519223 10.1093/hmg/ddm133

[b18] Novak A., Guo C., Yang W., Nagy A. & Lobe C. G. Z/EG, a double reporter mouse line that expresses enhanced green fluorescent protein upon Cre-mediated excision. Genesis 28, 147–155 (2000). 11105057

[b19] Srinivas S. . Cre reporter strains produced by targeted insertion of EYFP and ECFP into the ROSA26 locus. BMC Dev. Biol. 1, 4 (2001). 11299042 10.1186/1471-213X-1-4 PMC31338

[b20] Allen Mouse Brain Atlas Allen Institute for Brain Science. Allen Mouse Brain Atlas (online). Available from http://mouse.brain-map.org/.

[b21] Arlotta P., Molyneaux B. J., Jabaudon D., Yoshida Y. & Macklis J. D. Ctip2 controls the differentiation of medium spiny neurons and the establishment of the cellular architecture of the striatum. J. Neurosci. 28, 622–632 (2008). 18199763 10.1523/JNEUROSCI.2986-07.2008 PMC6670353

[b22] Matamales M. . Striatal medium-sized spiny neurons: identification by nuclear staining and study of neuronal subpopulations in BAC transgenic mice. PLoS ONE 4, e4770 (2009). 19274089 10.1371/journal.pone.0004770 PMC2651623

[b23] Minichiello L. . Essential role for TrkB receptors in hippocampus-mediated learning. Neuron 24, 401–414 (1999). 10571233 10.1016/s0896-6273(00)80853-3

[b24] Agoston D. V. . Ikaros is expressed in developing striatal neurons and involved in enkephalinergic differentiation. J. Neurochem. 102, 1805–1816 (2007). 17504264 10.1111/j.1471-4159.2007.04653.x

[b25] Durieux P. F. . D2R striatopallidal neurons inhibit both locomotor and drug reward processes. Nat. Neurosci. 12, 393–395 (2009). 19270687 10.1038/nn.2286

[b26] Bateup H. S. . Distinct subclasses of medium spiny neurons differentially regulate striatal motor behaviors. Proc. Natl Acad. Sci. USA 107, 14845–14850 (2010). 20682746 10.1073/pnas.1009874107 PMC2930415

[b27] Nestler E. J. Molecular basis of long-term plasticity underlying addiction. Nat. Rev. Neurosci. 2, 119–128 (2001). 11252991 10.1038/35053570

[b28] Hamers F. P., Lankhorst A. J., van Laar T. J., Veldhuis W. B. & Gispen W. H. Automated quantitative gait analysis during overground locomotion in the rat: its application to spinal cord contusion and transection injuries. J. Neurotrauma 18, 187–201 (2001). 11229711 10.1089/08977150150502613

[b29] Hamers F. P., Koopmans G. C. & Joosten E. A. CatWalk-assisted gait analysis in the assessment of spinal cord injury. J. Neurotrauma 23, 537–548 (2006). 16629635 10.1089/neu.2006.23.537

[b30] Vandeputte C. . Automated quantitative gait analysis in animal models of movement disorders. BMC Neurosci. 11, 92 (2010). 20691122 10.1186/1471-2202-11-92 PMC2924851

[b31] Bertran-Gonzalez J. . Opposing patterns of signaling activation in dopamine D1 and D2 receptor-expressing striatal neurons in response to cocaine and haloperidol. J. Neurosci. 28, 5671–5685 (2008). 18509028 10.1523/JNEUROSCI.1039-08.2008 PMC6670792

[b32] Hoover B. R. & Marshall J. F. Further characterization of preproenkephalin mRNA-containing cells in the rodent globus pallidus. Neuroscience 111, 111–125 (2002). 11955716 10.1016/s0306-4522(01)00565-6

[b33] Hoover B. R. & Marshall J. F. Population characteristics of preproenkephalin mRNA-containing neurons in the globus pallidus of the rat. Neurosci. Lett. 265, 199–202 (1999). 10327165 10.1016/s0304-3940(99)00251-7

[b34] Yan Z., Feng J., Fienberg A. A. & Greengard P. D(2) dopamine receptors induce mitogen-activated protein kinase and cAMP response element-binding protein phosphorylation in neurons. Proc. Natl Acad. Sci. USA 96, 11607–11612 (1999). 10500224 10.1073/pnas.96.20.11607 PMC18081

[b35] Cai G., Zhen X., Uryu K. & Friedman E. Activation of extracellular signal-regulated protein kinases is associated with a sensitized locomotor response to D(2) dopamine receptor stimulation in unilateral 6-hydroxydopamine-lesioned rats. J. Neurosci. 20, 1849–1857 (2000). 10684886 10.1523/JNEUROSCI.20-05-01849.2000 PMC6772914

[b36] Kulagowski J. J. . 3-((4-(4-Chlorophenyl)piperazin-1-yl)-methyl)-1H-pyrrolo-2,3-b-pyridine: an antagonist with high affinity and selectivity for the human dopamine D4 receptor. J. Med. Chem. 39, 1941–1942 (1996). 8642550 10.1021/jm9600712

[b37] Zuccato C. & Cattaneo E. Brain-derived neurotrophic factor in neurodegenerative diseases. Nat. Rev. Neurol. 5, 311–322 (2009). 19498435 10.1038/nrneurol.2009.54

[b38] Gorski J. A., Zeiler S. R., Tamowski S. & Jones K. R. Brain-derived neurotrophic factor is required for the maintenance of cortical dendrites. J. Neurosci. 23, 6856–6865 (2003). 12890780 10.1523/JNEUROSCI.23-17-06856.2003 PMC6740724

[b39] Cattaneo E., Zuccato C. & Tartari M. Normal huntingtin function: an alternative approach to Huntington's disease. Nat. Rev. Neurosci. 6, 919–930 (2005). 16288298 10.1038/nrn1806

[b40] Cepeda C. . Transient and progressive electrophysiological alterations in the corticostriatal pathway in a mouse model of Huntington's disease. J. Neurosci. 23, 961–969 (2003). 12574425 10.1523/JNEUROSCI.23-03-00961.2003 PMC6741903

[b41] Cepeda C., Wu N., Andre V. M., Cummings D. M. & Levine M. S. The corticostriatal pathway in Huntington's disease. Prog. Neurobiol. 81, 253–271 (2007). 17169479 10.1016/j.pneurobio.2006.11.001 PMC1913635

[b42] Dorner J. L. . Corticostriatal dysfunction underlies diminished striatal ascorbate release in the R6/2 mouse model of Huntington's disease. Brain Res. 1290, 111–120 (2009). 19616518 10.1016/j.brainres.2009.07.019 PMC2745264

[b43] Rosas H. D., Feigin A. S. & Hersch S. M. Using advances in neuroimaging to detect, understand, and monitor disease progression in Huntington's disease. NeuroRx 1, 263–272 (2004). 15717027 10.1602/neurorx.1.2.263 PMC534942

[b44] Maeda T. . Electrophysiological characteristic of corticoaccumbens synapses in rat mesolimbic system reconstructed using organotypic slice cultures. Brain Res. 1015, 34–40 (2004). 15223364 10.1016/j.brainres.2004.04.033

[b45] Graybiel A. M. & Rauch S. L. Toward a neurobiology of obsessive-compulsive disorder. Neuron 28, 343–347 (2000). 11144344 10.1016/s0896-6273(00)00113-6

[b46] Menalled L. . Decrease in striatal enkephalin mRNA in mouse models of Huntington's disease. Exp. Neurol. 162, 328–342 (2000). 10739639 10.1006/exnr.1999.7327

[b47] Guzman M. S. . Elimination of the vesicular acetylcholine transporter in the striatum reveals regulation of behaviour by cholinergic-glutamatergic co-transmission. PLoS Biol. 9, e1001194 (2011). 22087075 10.1371/journal.pbio.1001194 PMC3210783

[b48] Lobo M. K. . Cell type-specific loss of BDNF signaling mimics optogenetic control of cocaine reward. Science 330, 385–390 (2010). 20947769 10.1126/science.1188472 PMC3011229

[b49] Picconi B. . Plastic and behavioral abnormalities in experimental Huntington's disease: a crucial role for cholinergic interneurons. Neurobiol. Dis. 22, 143–152 (2006). 16326108 10.1016/j.nbd.2005.10.009

[b50] Kitabatake Y., Hikida T., Watanabe D., Pastan I. & Nakanishi S. Impairment of reward-related learning by cholinergic cell ablation in the striatum. Proc. Natl Acad. Sci. USA 100, 7965–7970 (2003). 12802017 10.1073/pnas.1032899100 PMC164696

[b51] Kravitz A. V. . Regulation of parkinsonian motor behaviours by optogenetic control of basal ganglia circuitry. Nature 466, 622–626 (2010). 20613723 10.1038/nature09159 PMC3552484

[b52] Higley M. J. & Sabatini B. L. Competitive regulation of synaptic Ca2+ influx by D2 dopamine and A2A adenosine receptors. Nat. Neurosci. 13, 958–966 (2010). 20601948 10.1038/nn.2592 PMC2910780

[b53] Gundersen H. J. . Some new, simple and efficient stereological methods and their use in pathological research and diagnosis. APMIS 96, 379–394 (1988). 3288247 10.1111/j.1699-0463.1988.tb05320.x

[b54] Schneider T. . Hypoactive phenotype combined with motor deficits in Gtf2ird1 null mouse model relevant to Williams syndrome. Behav. Brain Res. 233, 458–473 (2012). 22652393 10.1016/j.bbr.2012.05.014

[b55] Calabresi P. . Dopamine and cAMP-regulated phosphoprotein 32 kDa controls both striatal long-term depression and long-term potentiation, opposing forms of synaptic plasticity. J. Neurosci. 20, 8443–8451 (2000). 11069952 10.1523/JNEUROSCI.20-22-08443.2000 PMC6773171

