# Supplementary material for: BDNF–TrkB signaling in striatopallidal neurons controls inhibition of locomotor behavior
Source: Nat Commun. 2013 Jun 18;4:2031. doi: 10.1038/ncomms3031 (PMC3940866; doi:10.1038/ncomms3031)
Supplement: Supplementary Information — Supplementary Figures S1-S8, Supplementary Tables S1-S3, Supplementary Methods and Supplementary References [file ncomms3031-s1.pdf]

Supplementary Information

BDNF-TrkB Signaling in Striatopallidal Neurons Controls Inhibition of Locomotor Behaviour

Dario Besusso, Mirjam Geibel, Dana Kramer, Tomasz Schneider, Valentina Pendolino, Barbara Picconi, Paolo Calabresi, David M Bannerman and Liliana Minichiello

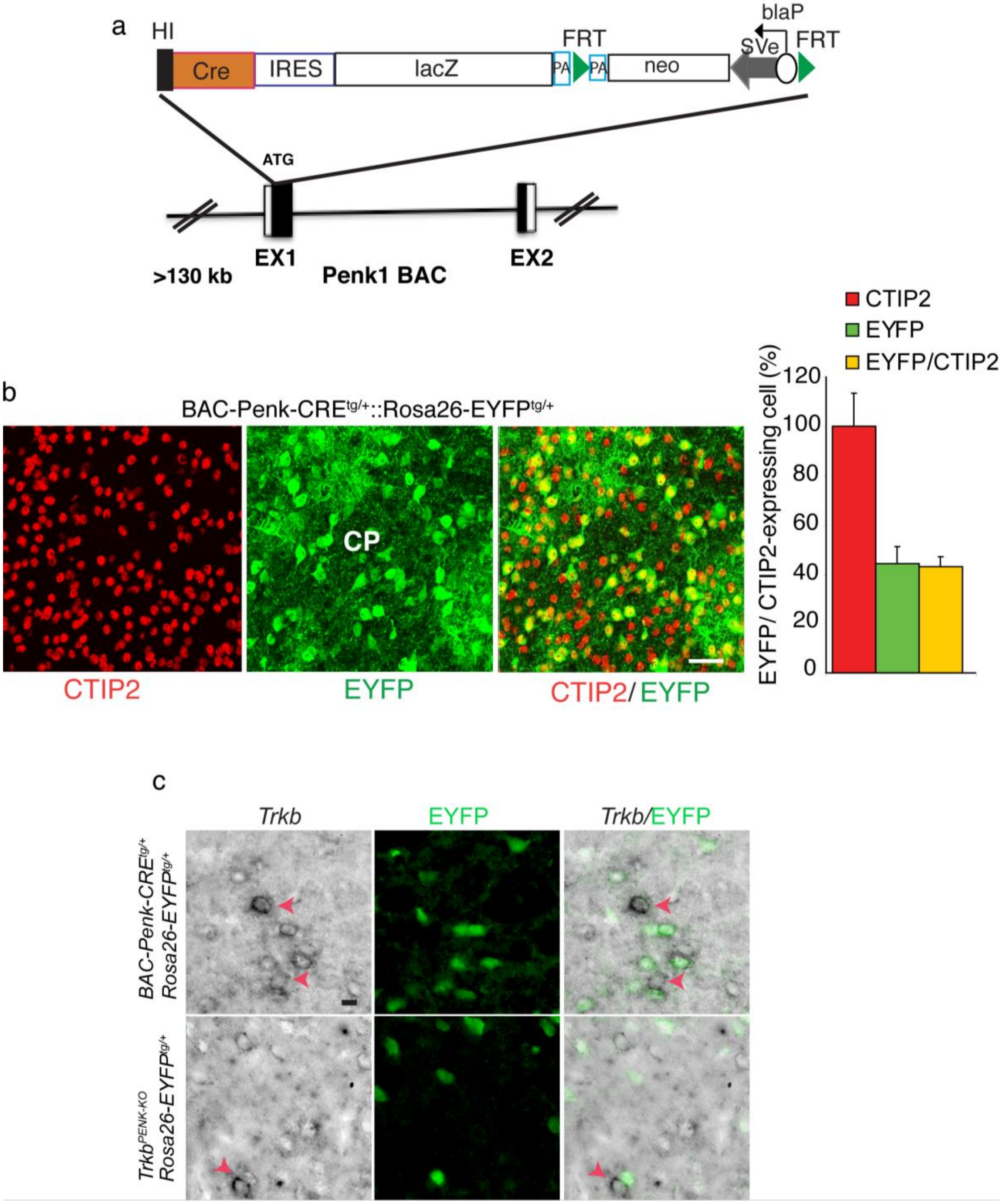
a
HI
Cre
IRES
lacZ
PA
FRT
PA
neo
blaP
SVe
FRT
ATG
EX1
EX2
>130 kb
Penk1 BAC
b
BAC-Penk-CRE$^{tg/+}$::Rosa26-EYFP$^{tg/+}$
CP
CTIP2
EYFP
CTIP2/ EYFP
CTIP2
EYFP
EYFP/CTIP2
EYFP/ CTIP2-expressing cell (%)
120
100
80
60
40
20
0
c
Trkb
EYFP
Trkb/EYFP
BAC-Penk-CRE$^{tg/+}$
Rosa26-EYFP$^{tg/+}$
Trkb$^{PENK-KO}$
Rosa26-EYFP$^{tg/+}$

Supplementary Figure S1. Generation of a *BAC-Penk-Cre* transgenic line. (a) A BAC containing the *Penk1* gene (Clone #RP24-289D15) was targeted via ET(RecE-RecT)-recombination[56] to insert a Cre-recombinase cassette into the ATG of the first coding exon of the gene. The cassettes inserted into the BACs consisted of a hybrid intron (HI) followed by the Cre-recombinase gene, an internal ribosomal entry site (IRES) followed by the beta-galactosidase gene (lacZ), to allow faster identification of transgene expression by X-gal staining, and a neomycin/kanamycin cassette, to allow selection of E.coli containing the modified BAC. After successful insertion of the transgene cassette into the BAC the neomycin/kanamycin cassette (flanked by FRT sites) was removed using Flp-recombinase. BAC DNA was prepared using a CsCl gradient centrifugation-based method or a QIAGEN kit for large constructs, the borders between the transgenic cassette and the BAC were sequenced to confirm the correct insertion of the Cre-IRES-lacZ cassette, circular BAC was then injected into mouse oocytes. (b) Representative images of a striatal field from *BAC-Penk-Cre*$^{tg/+}$*::Rosa26-eyfp*$^{tg/+}$ mice used for quantification of EYFP-expressing cells versus total striatal MSNs. Total MSNs were identified by the specific marker CTIP2. The percent of EYFP over CTIP2 (43.28 ± 3.77) is consistent with data previously reported[11]. Values are mean ±S.D. (c) Full-length *Trkb*-specific *in situ* hybridization of striatal tissue in control and mutant mice crossed with the reporter line Rosa26-EYFP (*BAC-Penk-Cre*$^{tg/+}$::*Rosa26-eyfp*$^{tg/+}$, and *Trkb*$^{PENK\text{-}KO}$::*Rosa26-eyfp*$^{tg/+}$, respectively). See also Supplementary methods. Images illustrate *Trkb* expression depleted in all EGFP+ cells in *Trkb*$^{PENK\text{-}KO}$, but retained in control sections and non-enkephalinergic cells. Red arrows indicate non-enkephalinergic expressing *Trkb* cells. OB, olfactory bulbs; CX, cortex; CP, caudate putamen; HP, hippocampus; CRB, cerebellum; NAc, nucleus accumbens; Scale bars: b, 500μm; c, 50μm; D, 10μm.

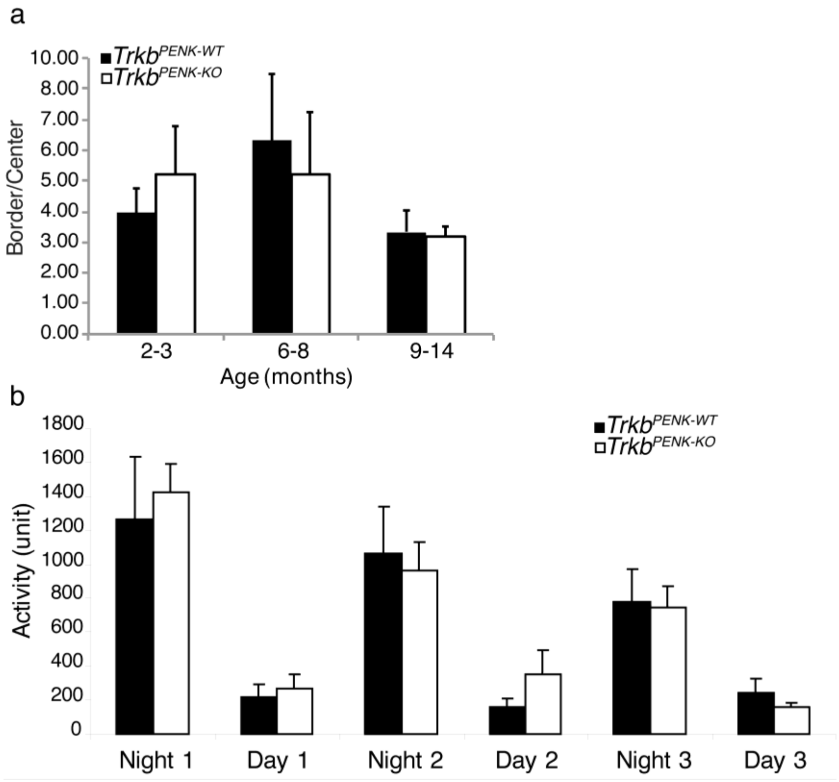

Supplementary Figure S2. Behavioural responses and basal activity are normal in *Trkb*$^{PENK\text{-}KO}$ mice. (a) Ratio of the time (measured in sec) spent in the border compared to the time spent in the center of the open field chamber during the assessment of spontaneous locomotor activity of controls and mutant mice (*Trkb*$^{PENK\text{-}KO}$, n= 9, 9, 7; *Trkb*$^{PENK\text{-}WT}$, n= 7, 9, 7, for the 3 age/groups, respectively) did not reveal any significant difference between the two genotypes analysed (Two-Way ANOVA, $F_{(1,42)}$=0.016, p=0.9006 main effect of genotype). Values are means ± S.E.M. (b) Basal activity was measured in 16M old control (n=6) and *Trkb*$^{PENK\text{-}KO}$ mice (n=4) by monitoring the mice for 3 days after being individually confined in their home-cages. Basal activity was detected by sensing the body-heat image in the infrared radiation; this analysis showed no differences between the two genotypes. Values are means ±S.E.M.

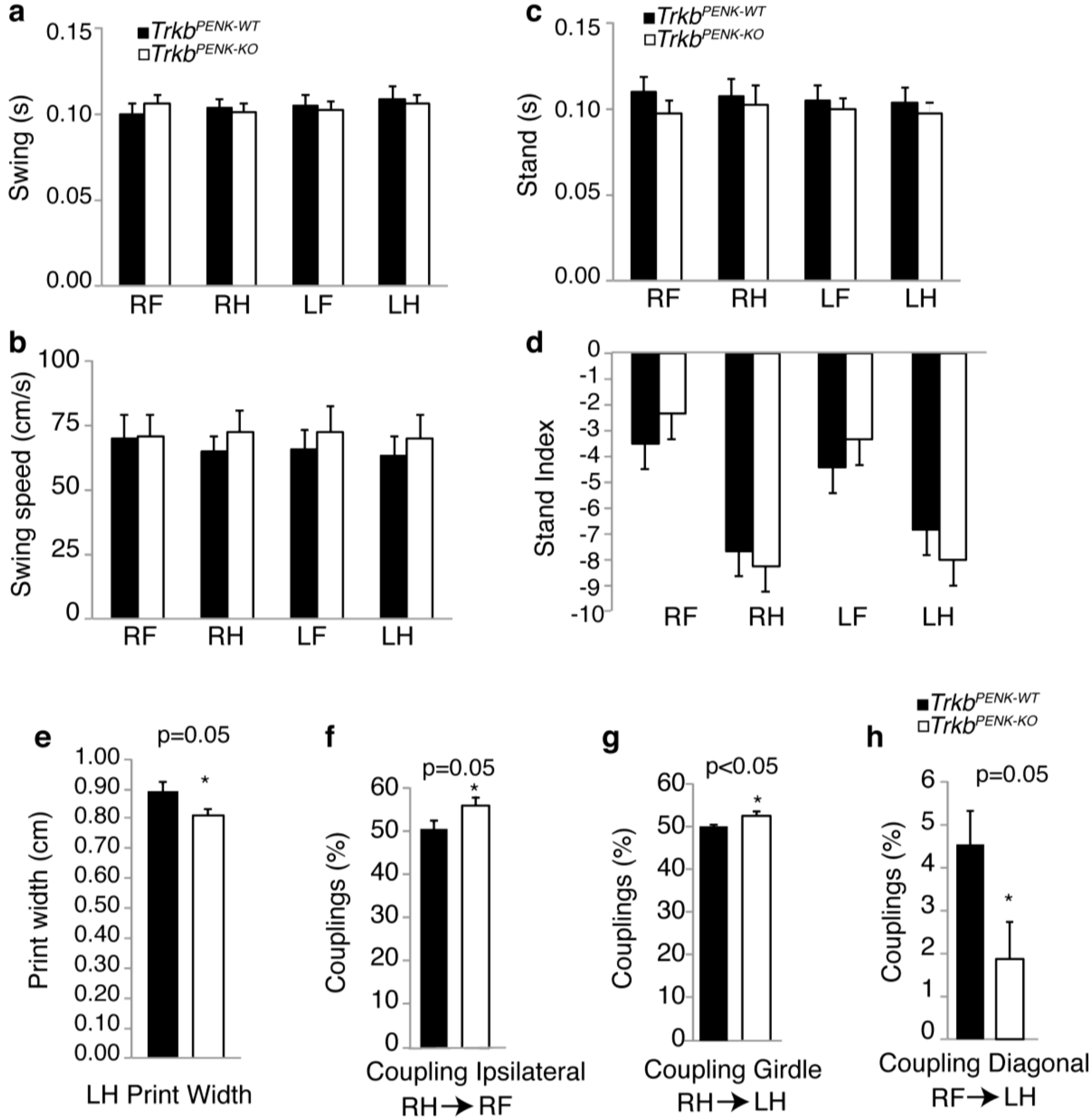

Supplementary Figure S3. Catwalk automated gait analysis parameters related to single paws and couplings in mice at 5M of age. (a-d) No differences between genotypes were found in dynamic paw parameters including (a) swing, (b) swing speed, (c) stand and (d) stand index. (e) Smaller print width for the left hind paw (LH) in *Trkb*$^{PENK\text{-}KO}$ mice compared to controls. (f-h) *Trkb*$^{PENK\text{-}KO}$ mice showed slightly decreased timely coordination between (f) ipsilateral (RH→RF) and (g) girdle paws (RH→LH), and better coordination between (h) diagonal paws (RF→LH) as measured by couplings. Values are means ± S.E.M.; n=5 per group, * $p<0.05$, two-tailed unpaired Student's t-test; RF – right forepaw, RH – right hindpaw, LF – left forepaw, LH – left hindpaw.

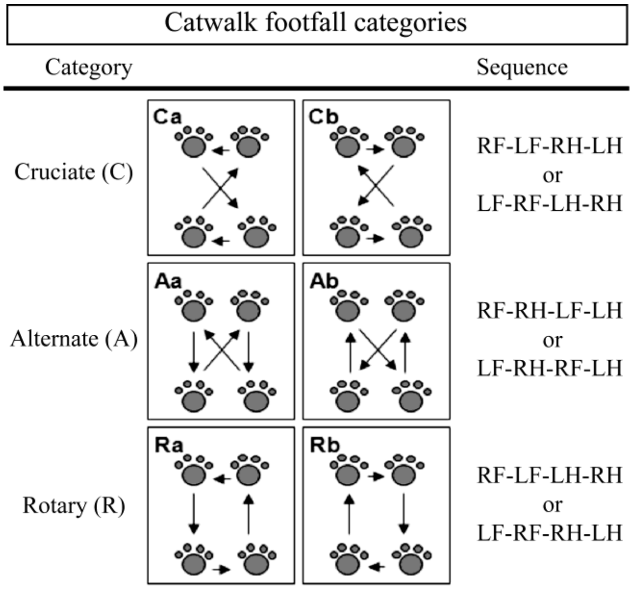

Supplementary Figure S4. Schematic representation of Catwalk footfall categories. LF – left front, LH – left hind, RH – right hind, RF – right front.

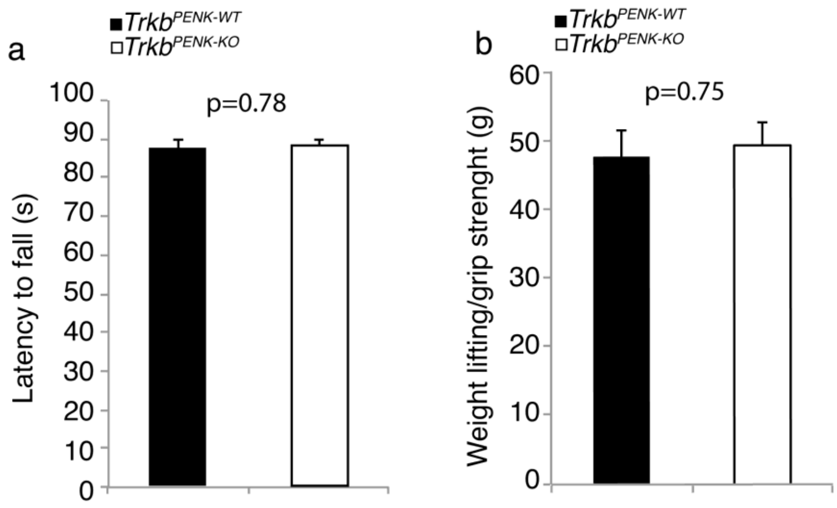

Supplementary Figure S5. Strength measured in 5M of age *Trkb*$^{PENK\text{-}KO}$ mice and control littermates by (a) inverted screen test and (b) weight lifting test. Values are means ± S.E.M.; n = 5 per group. P values were generated by two-tailed unpaired Student's t-test.

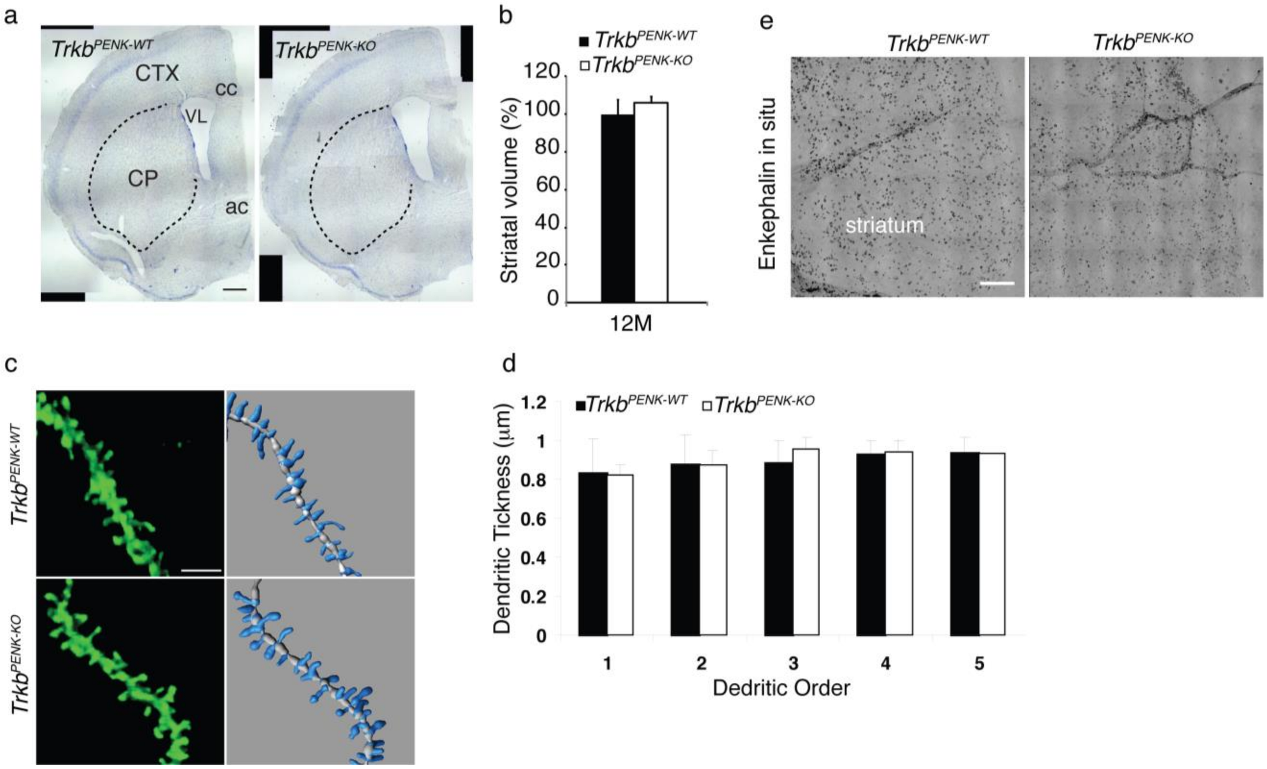

Supplementary Figure S6. Reduced striatal enkephalin expression despite intact morphology in absence of BDNF-TrkB signaling. (a) Representative images of coronal sections stained with cresyl violet showing different brain regions including the caudate putamem in *Trkb*$^{PENK\text{-}KO}$ and control mice at 12M of age. (b) Stereological measures revealed normal striatal volume in *Trkb*$^{PENK\text{-}KO}$ mice at 12M of age compared to controls (*Trkb*$^{PENK\text{-}WT}$, 6.51±0.53 mm^3, n=2; *Trkb*$^{PENK\text{-}KO}$, 6.89±0.24 mm^3, n=4; p= 0.25, two tailed unpaired Student's t-test; values are means ± S.D.). See also Supplementary methods. (c) Representative confocal images of Golgi-impregnated MSN dendrites and their computer reconstructions. (d) Confocal stacks of Golgi-impregnated MSNs from 12M old mice were analyzed for dendritic thickness (n=30, 10 randomly selected neurons per mouse/over 3 mice). Values are means ± S.E.M. (e) Representative sections from 18M old control and *Trkb*$^{PENK\text{-}KO}$ mice showing an overview of the striatal enkephalin in situ hybridization. Mutants show an obvious reduction in enkephalin expression. Scale bars, a, 500µm; c, 30µm; e, 200µm.

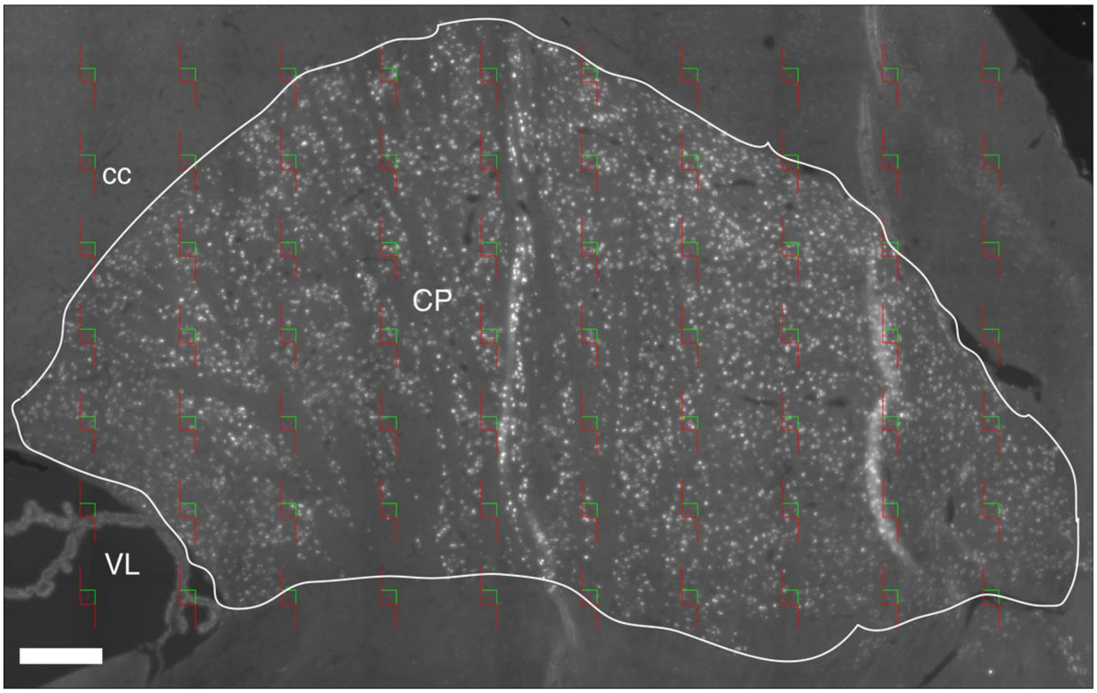

Supplementary Figure S7. **Stereological estimation of cell density.** Image of typical sagittal section of mouse brain used for the stereological estimation of DARPP32 positive cells as an example. Cells were counted with an unbiased counting frame size 50x30 μm for DARPP32. The frames were randomly positioned by the "Unbiased counting frame" macro of ImageJ (v1.46r, NIH) with a regular distance between frames of x=300, y=280 μm. Cells in each frame were counted complying with the optical fractionator counting rules. Striatal boundaries were defined using the following morphological references: the corpus callosum, the external capsule, the lateral ventricle and the anterior commissure. Scale bar, 300 μm. CP, caudate putamen; VL, lateral ventricle; cc, corpus callosum.

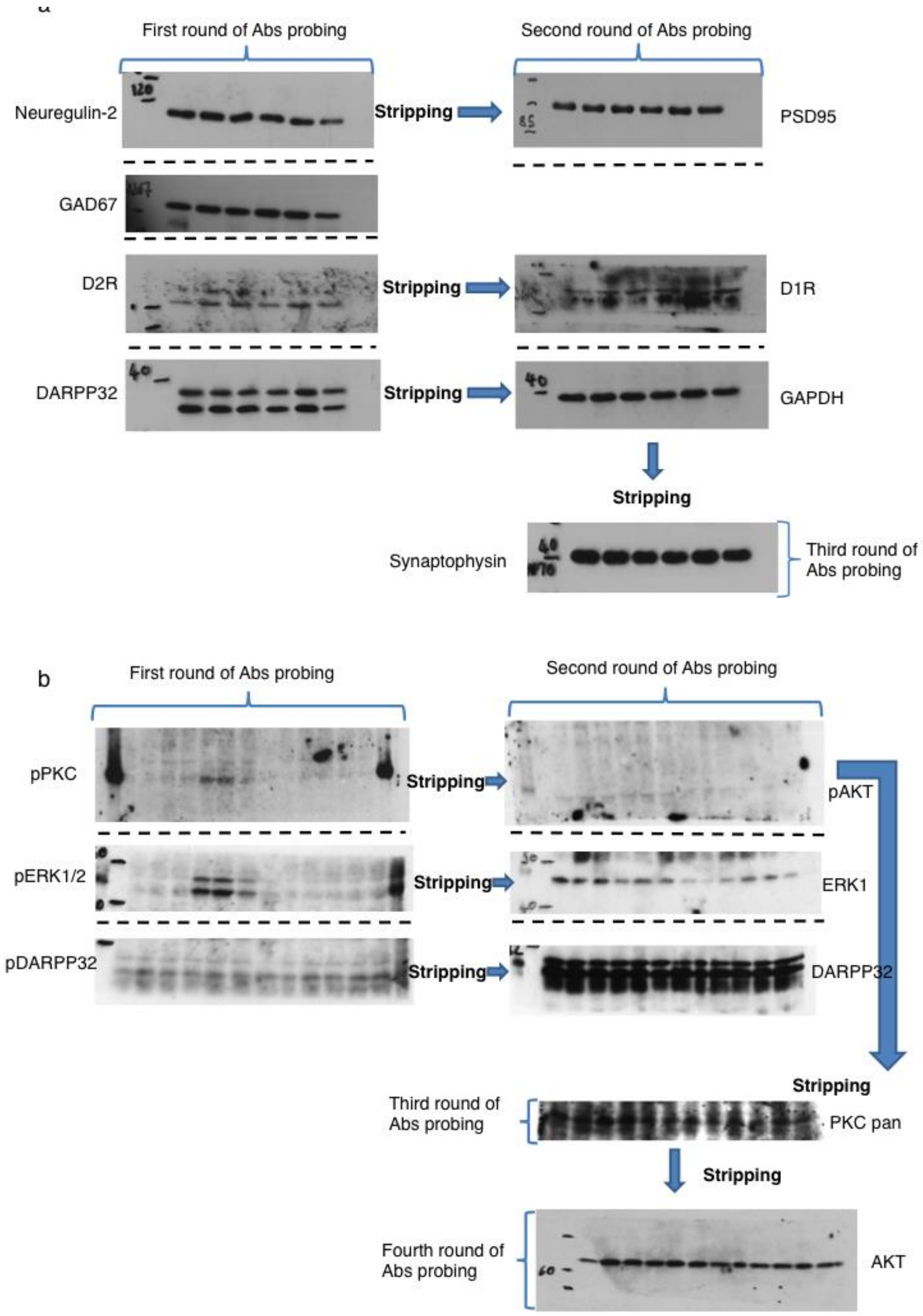
a
First round of Abs probing
Second round of Abs probing
Neuregulin-2
Stripping
PSD95
GAD67
D2R
Stripping
D1R
DARPP32
Stripping
GAPDH
Stripping
Synaptophysin
Third round of Abs probing
b
First round of Abs probing
Second round of Abs probing
pPKC
Stripping
pAKT
pERK1/2
Stripping
ERK1
pDARPP32
Stripping
DARPP32
Stripping
Third round of Abs probing
PKC pan
Stripping
Fourth round of Abs probing
AKT

c

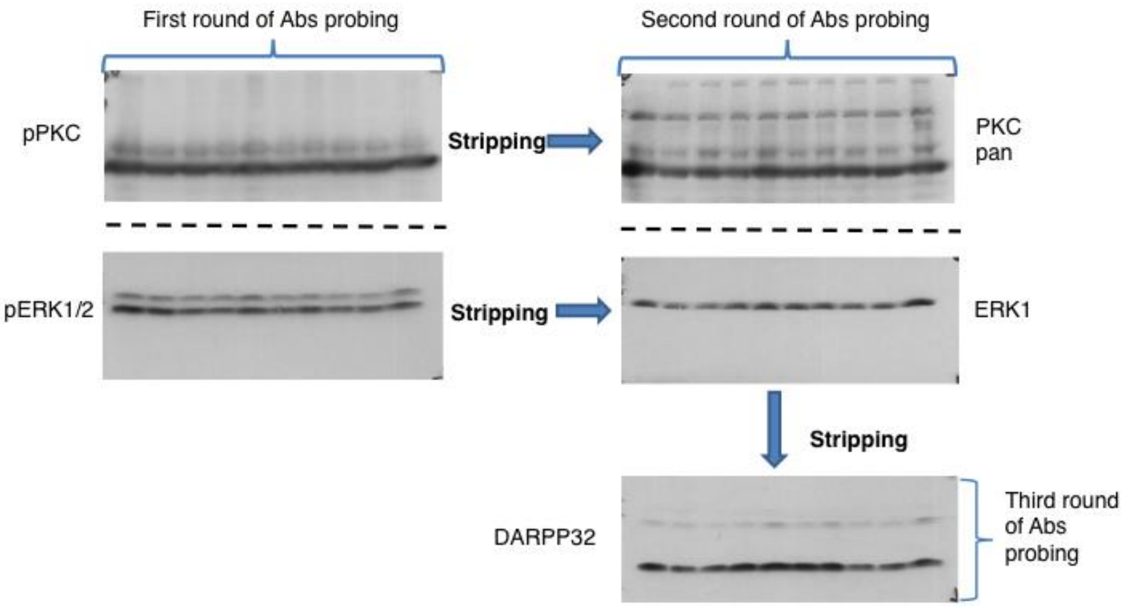

Supplementary Figure S8. Representative original gels.
(a-c) Blots showed in the figures (3e, 7a and 7b, respectively) come from multiple probing of the same membrane. In order to do so, the membranes obtained upon transfer of a long mini gel were horizontally cut around the expected molecular weight of the target protein (this was determined by probing an independent whole membrane with the antibody of interest). Each membrane was cut into 3 to 4 pieces and incubated with the appropriate antibody. In the case of figure 7, pospho-specific antibodies were probed first in order to avoid any alteration of the signal that may result from the stripping procedure. Once the appropriate band was detected and analyzed membranes were stripped by low-pH stripping buffer, re-exposed to control for successful stripping, and then probed again with the non-pospho antibody. Each gel was repeated 2 to 3 times to ensure consistency of the data. Dashed lines indicate the region where the membrane was cut.

Supplementary Table S1

General gait parameters are normal in *Trkb*$^{PENK\text{-}KO}$ mice compared to controls.	*Trkb*$^{PENK\text{-}WT}$		*Trkb*$^{PENK\text{-}KO}$	
	mean	SD	mean	SD
Run_Duration_(s)_Mean	1.96	0.41	1.72	0.42
Average_Speed_(cm/s)_Mean	32.05	11.23	36.10	10.81
Maximum_Speed_Variation_(%)_Mean	21.80	4.37	17.13	4.37
Number Of Steps	84.60	12.44	79.00	13.47
Cadence	19.30	3.43	19.70	2.55
BOS_FrontPaws_(cm)_Mean	1.06	0.15	0.94	0.09
BOS_HindPaws_(cm)_Mean	2.05	0.16	1.94	0.23
Stride Length_(cm)_Mean_HindPaws	6.29	1.07	6.96	1.30
Stride Length_(cm)_Mean_FrontPaws	6.33	1.08	6.95	1.33

**Supplementary Table S2** Single paw parameters are not significantly different between mutants and controls.	*Trkb*$^{PENK-WT}$ mean	*Trkb*$^{PENK-WT}$ SD	*Trkb*$^{PENK-KO}$ mean	*Trkb*$^{PENK-KO}$ SD
RF_Stand_(s)_Mean	0.10	0.01	0.09	0.01
RF_MaxContactArea_(cm²)_Mean	0.65	0.11	0.58	0.10
RF_PrintLength_(cm)_Mean	1.03	0.09	0.98	0.05
RF_PrintWidth_(cm)_Mean	0.95	0.09	0.93	0.10
RF_MeanIntensity_Mean	139.60	6.52	135.54	4.46
RF_Swing_(s)_Mean	0.09	0.01	0.10	0.01
RF_SwingSpeed_(cm/s)_Mean	69.92	19.33	70.13	18.85
RF_DutyCycle_(%)_Mean	52.37	4.34	47.10	4.65
RH_Stand_(s)_Mean	0.10	0.02	0.10	0.02
RH_MaxContactArea_(cm²)_Mean	0.71	0.10	0.66	0.08
RH_PrintLength_(cm)_Mean	1.11	0.05	1.10	0.05
RH_PrintWidth_(cm)_Mean	0.89	0.10	0.84	0.07
RH_MeanIntensity_Mean	158.09	3.86	157.40	5.86
RH_Swing_(s)_Mean	0.10	0.01	0.10	0.01
RH_SwingSpeed_(cm/s)_Mean	64.39	13.03	72.55	17.57
RH_DutyCycle_(%)_Mean	49.71	6.67	48.66	6.45
LF_Stand_(s)_Mean	0.10	0.02	0.09	0.01
LF_MaxContactArea_(cm²)_Mean	0.63	0.11	0.61	0.10
LF_PrintLength_(cm)_Mean	1.02	0.09	1.01	0.08
LF_PrintWidth_(cm)_Mean	0.90	0.07	0.93	0.10
LF_MeanIntensity_Mean	142.01	9.49	137.00	4.11
LF_Swing_(s)_Mean	0.10	0.01	0.10	0.01
LF_SwingSpeed_(cm/s)_Mean	65.20	16.85	72.40	21.21
LF_DutyCycle_(%)_Mean	49.18	4.36	48.75	2.34
LH_Stand_(s)_Mean	0.35	0.93	0.71	0.50
LH_MaxContactArea_(cm²)_Mean	0.68	0.10	0.62	0.07
LH_PrintLength_(cm)_Mean	1.08	0.06	1.06	0.04
LH_PrintWidth_(mm)_Mean	0.17	0.89	0.05	0.46
LH_MeanIntensity_Mean	158.12	2.19	155.78	6.16
LH_Swing_(s)_Mean	0.10	0.01	0.10	0.01
LH_SwingSpeed_(mm/s)_Mean	63.22	15.39	69.91	20.89

RF – right forelimb, RH – right hindlimb, LF – left forelimb, LH – left hindlimb.

Supplementary Table S3

Coordination-related parameters are not significantly different in *Trkb*$^{PENK-KO}$ compared to controls.	*Trkb*$^{PENK-WT}$ mean	*Trkb*$^{PENK-WT}$ SD	*Trkb*$^{PENK-KO}$ mean	*Trkb*$^{PENK-KO}$ SD
StepSequence_RegularityIndex (RI)_(%)	98.79	0.82	98.65	1.01
StepSequence_NumberOfPatterns	19.60	2.70	17.60	3.20
*StepSequence_CA_(%)	14.37	12.46	17.92	15.93
*StepSequence_CB_(%)	34.63	12.92	17.53	13.60
*StepSequence_AA_(%)	42.06	17.50	58.99	25.50
*StepSequence_AB_(%)	8.92	9.94	5.54	7.22
Couplings_RF->LH_Mean	4.52	1.78	1.86	1.89
Couplings_LF->RH_Mean	2.39	2.21	0.82	0.83
Couplings_LH->RF_Mean	5.90	2.87	5.01	2.94
Couplings_RH->LF_Mean	6.79	2.41	5.91	3.54
Couplings_LH->RH_Mean	47.95	1.85	47.28	1.85
Couplings_LF->RF_Mean	47.72	4.14	49.79	2.30
Couplings_RH->LH_Mean	49.97	0.66	52.40	1.76
Couplings_RF->LH_Mean	50.89	3.62	49.29	2.49
Couplings_RF->RH_Mean	48.39	3.99	44.66	4.33
Couplings_LF->LH_Mean	48.64	3.35	46.65	3.38
Couplings_RH->RF_Mean	50.41	3.53	55.76	4.03
Couplings_LH->LF_Mean	50.85	3.48	53.23	4.31
PhaseDispersions_RF->LH_Mean	0.18	5.10	-3.79	4.85
PhaseDispersions_LF->RH_Mean	-2.63	2.48	-4.23	2.88
PhaseDispersions_LH->RH_Mean	47.13	6.23	47.85	1.81
PhaseDispersions_LF->RF_Mean	48.29	4.11	49.19	4.15
PhaseDispersions_RF->RH_Mean	48.51	3.98	44.66	4.33

*Diagrams illustrating the different regular step patterns[29]: cruciate, alternate and rotate. CA, Cruciate a; CB, Cruciate b; AA, Alternate a; AB, Alternate b; RA, Rotary a, RB, Rotary b. RF – right forelimb, RH – right hindlimb, LF – left forelimb, LH – left hindlimb.

Supplementary Methods

CatWalk quantitative gait analysis test

Detailed analysis of gait was performed using the CatWalk system (Noldus, the Netherlands) according to the previously published method[28,29,54].

General parameters:

Average speed [mm/s]

Maximum variation [%]: maximum variation in running speed

Cadence: number of steps per second

Parameters related to single paws:

Intensity of the paw print [arbitrary units]: mean intensity of the pixels forming the paw print

Maximum contact area [mm^2]: the paw area at the moment of maximal paw-floor contact during stance

Print area [mm^2]: the total floor area contacted by the paw during the stance phase; *Print/box width* [mm]: the width of the print area

Print/box length [mm]: the length of the print area

Print position/relative paw placement [mm]: the distance between the placement of the fore and hind paws of the same side of the animal at each step cycle

Stride length [mm]: the distance between two consecutive paw placements

Stand/stance phase duration [s]: the time of contact of one paw with the floor during a single step cycle

Swing [s]: the duration of no contact of a paw with the glass plate

Swing speed [m/s]: this parameter is computed from stride length and swing duration

Step cycle duration [s]: the time between two consecutive paw placements (stance + swing duration)

Duty factor [%]: the ratio between the stance duration and the step cycle duration [stance phase duration/(stance + swing duration) × 100]

Base of support (BOS) [mm]: mean distance between either the front paws or the hind paws measured perpendicular to the direction of walking and calculated per run)

Parameters related to the interlimb coordination:

Step sequence distributions: the order in which the paws are placed on the glass plate described in Supplementary Table S3.

The regularity index (RI) [%]: the number of normal step sequence patterns relative to the total number of paw placements [RI = (NSSP x 4/PP) x 100%], where NSSP represents the number of normal step sequence patterns and PP the total number of paw placements

The average phase lag and the phase lag variability: the time of initial contact of one paw (the target) expressed as a percentage to the stride length of another paw (the anchor), phase lags can be calculated between the paws of the same girdle (forepaws or hindpaws), between paws on the same side (ipsilateral left or ipsilateral right), and between diagonal paws (opposite forepaw/hindpaw)

Coupling: the temporal relationship between placements of two paws within step cycle computed the same way as phase lags.

Data analysis was performed by automatically labelling all areas containing one or more pixels above a 25 pixel threshold and then assigning those areas to one of the paws. A typical run/crossing contained at least six step cycles and averaged data from all step cycles in a run were used in the analysis.

***In-situ* hybridization**: RNA probes were prepared by PCR (*Trkb*: forward 5' TCAGCATATCAAGAGACAC, reverse 5' CTGTACACATCTCGGGACAT; *Enkephalin*: Allen Mouse Brain Atlas probes, Riboprobe ID: RP_060315_01_A07, http://mouse.brain-map.org). Cryosections were washed with PBS, treated with 10 µg ml^{-1} Proteinase K (Roche) in PBS for 6 min at 37°C, fixed in 4%(wt/vol) PFA for 10 min and washed with PBS. Acetylation was performed for 10 min in 0.25% acetic anhydride, prehybridized and then hybridized with biotinylated RNA probes (800 ng ml^{-1}) at 58°C for 24-40 hours. Post-hybridization washes consisted of 30 min 2x SSC at room temperature, 2 times 30 min 2x SSC at 65°C, 2 times 30 min 0.1 SSC. After blocking, sections were incubated with an alkaline phosphatase-conjugated DIG antibody (Roche) and staining was developed with BCIP/NBT. When immunofluorescence was performed, sections were refixed 30 min in 4%(wt/vol) PFA after the last PBS rinse, and staining was performed as described above.

Striatal volume: Striatal volume was estimated based on stereological analysis of 16-18 rostral to caudal coronal sections for each mouse. Striatal boundaries were defined on Nissl-stained sections using the following morphological references: the corpus callosum, the external capsule, the lateral ventricle and the anterior commissure. The volume was reconstructed and quantified using the software Reconstruct[57].

Morphological analysis of MSNs: Tissues were embedded in 5% gelatin (Sigma) and sectioned sagitally with a thickness of 150 µm using a vibratome (Leica). Golgi impregnation was performed using the Rapid GolgiStain Kit (FD NeuroTechnologies) according to the manufacturer instructions. Fully impregnated striatal medium spiny neurons were identified by their morphology and by the presence of dendritic spines. Brains from 3 mutants and 3 controls were used for this analysis. Ten neurons from each brain showing dendritic arborization without obvious truncations were analyzed by confocal microscopy using 63x/0.8 NA objective. The neuron tracing software (Imaris, Bitplane version 7.1.1) was then used to reconstruct the single neuron morphology by a semi-automated procedure. Specifically, the method relies on an automated procedure exclusively for the reconstruction of the cell soma volume and dendritic arborization but spines were selected manually from confocal z-stacks and considered such when fully visible with clearly identifiable head. A threshold for the minimum spine head diameter was set at ≥0.143 µm in order to avoid the identification of false positive. Moreover, the branch length was >0.5 µm. The Imaris software calculated then spine density, dendritic thickness and complexity of the dendritic arborization. These parameters were chosen according to previously published procedure based on the same software[58,59]. For the analysis of punctae images of double immunofluorescent staining from 3 $Trkb^{PENK\text{-}WT}$ and 3 $Trkb^{PENK\text{-}KO}$ mice obtained with specific antibodies were analysed by confocal microscopy. Punctae on each parvalbumin stained cell were then selected when present at the cell surface with a minimum diameter of 0.2µm for gephyrin and 0.5 µm for GAD67. For each animal 8 to 10 cells were acquired. The punctae density was then calculated by Imaris software according to previously published work[58,59].

cFos-ir and punctate labelling quantification: Counts of cFos-ir cells in the CPu and LGP were performed on 5 to 6 non-consecutive medial to lateral sagittal sections (spanning around 600 μm). LGP location was determined based on the expression of the marker parvalbumin that is first of all expressed within the LGP and secondly, strongly highlights the reticular nucleus of the thalamus that dorso-laterally defines the edge of the globus pallidus. A surface of approximately 1 to 2 mm^2 for the CPu and of 0.1 to 0.4 mm^2 for the LGP was acquired by tile scan imaging from each section. The density of cFos-ir cells in the specified area was then determined by counting the total number of neurons in the area using the software Imaris (Bitplane). Adjacent sections were then used to determine the density of GAD67- and gephyrin-containing synapses on pallidosubthalamic LGP neurons. Cells were identified within the LGP by parvalbumin labeling. The parvalbumin staining was also used to determine the volume of the soma and the first tract of the primary dendrites (when visible). Punctate labeling corresponding to the surface of the parvalbumin staining was analyzed by the use of the software Imaris. The density of sphere-shaped positive area for GAD67 and gephyrin was determined. Minimum of 5 to 8 cells per mouse were analyzed (3 animals per genotype).

Electrophysiological experiments: Brain slices (200-300μm) from tissue blocks were prepared with a vibratome. Single slices was transferred to a recording chamber and submerged in a continuously flowing Krebs' solution (33°C, 2–3 ml min^{-1}) gassed with 95%(vol/vol) O2 and 5%(vol/vol) CO2. Whole-cell recordings were performed using 1.5 mm external diameter borosilicate pipettes (Harvard Apparatus, USA). For spontaneous postsynaptic currents (sEPSCs), in voltage clamp mode, pipettes were filled with (in mM): 120 CsMeSO3, 15 CsCl, 8 NaCl, 0.2 EGTA, 10 HEPES, 2 Mg-ATP, 0.3 Na-GTP, 10 tetraethylammonium, and 5 QX-314, pH7.2, with CsOH. Access resistances were <20 MΩ. Membrane current was filtered at 0.1 kHz and digitized at 100–200 μs using Clampex 10.2 (gap-free mode). Cells were voltage-clamped at −70 mV to determine basic membrane properties and to examine spontaneous EPSCs. Picrotoxin (50μm) was applied to block $GABA_A$ receptor-mediated currents. Baseline activity was recorded for 3–5 min after 10-12 min in picrotoxin. For extracellular recordings borosilicate pipettes were filled with 2 M NaCl (resistance 10–15 MΩ). Amplitude of input-output responses were plotted and fit by a

sigmoidal function and then compared for statistical significance using Prism software. Testing stimuli of 0.1 Hz, 250-300μs duration and 20-30V amplitude were chosen to evoke field EPSPs that were 80-90% of maximum amplitude. Paired-pulse ratio indexes were calculated as mean ratio of response amplitude (amplitude of 2_{nd}fEPSP/1_{st}fEPSP) to paired-pulse stimulation (PPS) at 40 and 60 ms interpulse intervals.

Supplementary References

56 Muyrers, J. P. P., Zhang, Y., Benes, V., Testa, G., Rientjes, J.M., Stewart, A.F. ET Recombination, DNA Engineering Using Homologous Recombination in E. coli. *Methods in Molecular Biology* **256**, 107-121 (2004).

57 Fiala, J. C. Reconstruct: a free editor for serial section microscopy. *J Microsc.* 218, 52-61 (2005).

58 Shen, H., Sesack, S,R., Toda, S., Kalivas, P,W. Automated quantification of dendritic spine density and spine head diameter in medium spiny neurons of the nucleus accumbens. *Brain Struct Funct.* 213, 149-157 (2008).

59 Shen, H., W., Toda, S., Moussawi, K., Bouknight, A., Zahm, D,S., Kalivas, P,W. Altered dendritic spine plasticity in cocaine-withdrawn rats. *J Neurosci.* 29, 2876-2884 (2009).
